# The Challenge of Lyssavirus Infections in Domestic and Other Animals: A Mix of Virological Confusion, Consternation, Chagrin, and Curiosity

**DOI:** 10.3390/pathogens14060586

**Published:** 2025-06-13

**Authors:** Charles E. Rupprecht, Aniruddha V. Belsare, Florence Cliquet, Philip P. Mshelbwala, Janine F. R. Seetahal, Vaughn V. Wicker

**Affiliations:** 1 College of Forestry, Wildlife and Environment, Auburn University, Auburn, AL 36849, USA; avb0033@auburn.edu; 2 College of Veterinary Medicine, Auburn University, Auburn, AL 36849, USA; 3 EU/WOAH/WHO Reference Laboratory for Rabies, OMCL for Rabies Vaccines, 54220 Malzéville, France; florence.cliquet@anses.fr; 4 NSW Department of Primary Industries, Orange, NSW 2800, Australia; p.mshelbwala@uq.net.au; 5 Department of Diagnostic Medicine/Pathobiology, College of Veterinary Medicine, Kansas State University, Manhattan, KS 66502, USA; jseetahal@vet.k-state.edu; 6 Independent Researcher, Atlanta, GA 30307, USA; vaughn.v.wicker@gmail.com

**Keywords:** challenges, diagnostics, epidemiology, lyssavirus, One Health, rabies, vaccines, veterinary, virology, zoonoses

## Abstract

Lyssaviruses are RNA viruses in the Family Rhabdoviridae, Genus *Lyssavirus*. They represent the causative agents of acute, progressive encephalitis, known historically as rabies. Regardless of specific etiology, their collective viral morphology, biochemistry, pathobiology, associated clinical signs, diagnosis, epizootiology, and management are essentially the same. Despite centuries of clinical recognition, these quintessential neurotropic agents remain significant pathogens today, with substantive consequences to agriculture, public health, and conservation biology. Notably, the singular morbidity caused by lyssaviruses is incurable and constitutes the highest case fatality of any viral disease. All warm-blooded vertebrates are believed to be susceptible. The dog is the only domestic animal that serves as a reservoir, vector, and victim. In contrast, felids are effective vectors, but not reservoirs. All other rabid domestic species, such as livestock, constitute spillover infections, as a bellwether to local lyssavirus activity. Frequently, professional confusion abounds among the veterinary community, because although the viral species *Lyssavirus rabies* is inarguably the best-known representative in the Genus, at least 20 other recognized or putative members of this monophyletic group are known. Frequently, this is simply overlooked. Moreover, often the ‘taxonomic etiology’ (i.e., ‘*Lyssavirus x*’) is mistakenly referenced in a biopolitcal context, instead of the obvious clinical illness (i.e., ‘rabies’). Global consternation persists, if localities believe they are ‘disease-free’, when documented lyssaviruses circulate or laboratory-based surveillance is inadequate to support such claims. Understandably, professional chagrin develops when individuals mistake the epidemiological terminology of control, prevention, elimination, etc. Management is not simple, given that the only licensed veterinary and human vaccines are against rabies virus, sensu lato. There are no adequate antiviral drugs for any lyssaviruses or cross-reactive biologics developed against more distantly related viral members. While representative taxa among the mammalian Orders Chiroptera, Carnivora, and Primates exemplify the major global reservoirs, which mammalian species are responsible for the perpetuation of other lyssaviruses remains a seemingly academic curiosity. This zoonosis is neglected. Clearly, with such underlying characteristics as a fundamental ‘disease of nature’, rabies, unlike smallpox and rinderpest, is not a candidate for eradication. With the worldwide zeal to drive human fatalities from canine rabies viruses to zero by the rapidly approaching year 2030, enhanced surveillance and greater introspection of the poorly appreciated burden posed by rabies virus and diverse other lyssaviruses may manifest as an epidemiological luxury to the overall global program of the future.

## 1. Introduction

The origins of an often-repeated quote on infectious diseases remain speculative (i.e., “…it is time to close the book on infectious diseases and declare the war against pestilence won...”), but writ large, the ‘microbial war’ has only begun [1]. In human and veterinary medicine, ‘lists’ of infectious diseases pose a challenge to the fresh graduate and the seasoned professional. This is due in part to evolution, climate change, environmental degradation, globalization, technology, the infodemic, etc. [2,3,4,5,6].

Broadly considered, such pathogens (i.e., from the Greek ‘suffering producer’) run a diverse epidemiological gamut: in recognition, over millennia to newly emergent; in occurrence, from frequent to rare; in distribution, from worldwide to local; in phylogenetic host breadth, from broad to narrow; in morbidity, from mild to severe; in biomedical response, from easily identified and treatable to diagnostic dilemmas and the incurable; in political support, from prioritization to neglected; in academic parlance, from the commonly taught to the nearly obscure; in global significance, from the simmering pandemic potential to the quickly forgotten, extinct phenomenology; and everything in between [7,8,9,10,11]. Transdisciplinary, One Health facets, particularly concerning threats to individual biosafety, interspecific communicability, food security, health disparities, veterinary economics, zoonotic risks, biopolitics, and other subjective criteria, help to justify topical inclusion within the basic student curriculum space and broaden the need for continuing professional training [12,13,14,15,16,17,18].

Considering this mélange, lyssaviruses are ideal viral pathogens for discussion within the somewhat complicated and challenging microbiological realm. Lyssaviruses are the singular etiological agents of the same disease, known historically as rabies (i.e., to rage). In essence, these pathogens are distributed globally, where diverse mammalian reservoirs pose threats of viral exposure to humans, domestic animals, and wildlife. Moreover, despite this same disease being recognized for millennia, its discrete and variable causation at a taxonomic level was identified less than 50 years ago, creating considerable confusion, consternation, chagrin, and curiosity.

The aim of our review is to provide a scientific perspective of the challenges faced in modern veterinary medicine by lyssaviruses at a global level, illustrated by selected case examples, and supported by key historical citations and relevant articles in the peer-reviewed literature [1–538].

## 2. Etiological Agents and Taxonomy

**Figure 1:**
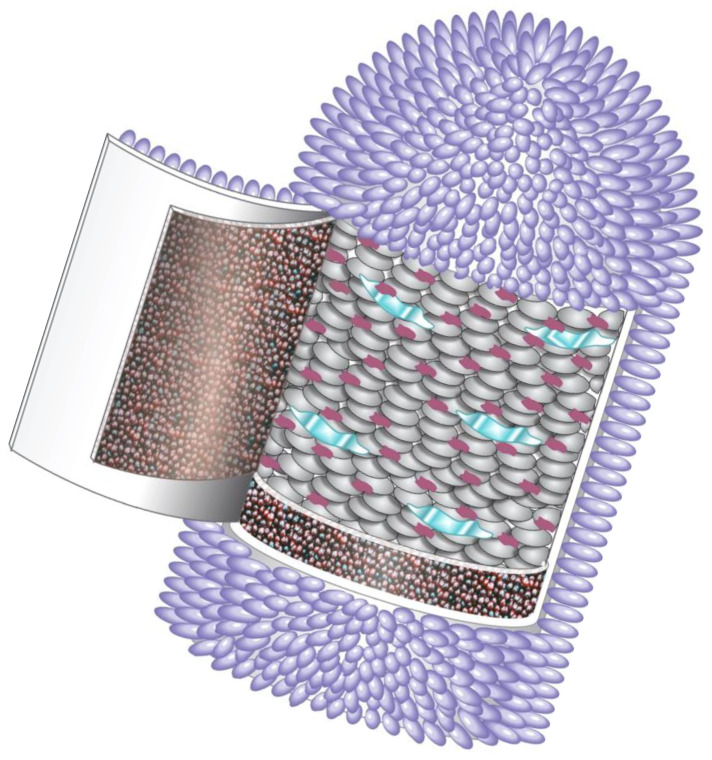
Conception of a generalized bullet-shaped lyssavirus virion, illustrating the outer G protein spikes (purple), extending through the host-cell derived membrane (white), with a cut-away view of the M protein (black and red beaded stippling) encasing the helical ribonucleoprotein core, consisting of the N protein (gray) encapsidated to the single strand of RNA, the P protein (magenta), and the L protein (teal), also known as the RNA-dependent RNA polymerase (Courtesy I. Kuzmin, University of Texas Medical Branch, Galveston, TX, USA).

Part of the potential confusion in veterinary medicine over disease (e.g., rabies) and etiology (e.g., lyssavirus) lies in an appreciation of modern taxonomy (i.e., from the Greek, ‘order’ or arrangement’ and ‘method’ or ‘law’) applied to the field of virology. While organisms, such as mammals (e.g., a domestic carnivore, the dog, Family Canidae, *Canis familiaris*), are categorized and arranged taxonomically due to shared traits (ideally reflective of evolutionary relationships), viruses are classified into various groups, essentially based upon structure and biochemical properties, using the practice of binomial nomenclature. Overall intentions of viral taxonomy were towards the achievement of greater uniformity, recognition, communication, and clarity in the field [19]. Lyssaviruses are rod-like, single-stranded, negative-sense RNA viruses. Taxonomically, they represent a distinct, monophyletic group, residing in the Family Rhabdoviridae, Genus *Lyssavirus*, with demarcation criteria based in part upon nucleotide identity, serology, and niche space [20]. All lyssavirus virions possess a similar bullet-shaped morphology (~60–110 nm × ~130–250 nm). The nucleic acid is contained within an inner, helical nucleocapsid, with an outer, membrane-bound protein coat (Figure 1).

Five monocistronic genes encode the structural proteins, consisting of a nucleoprotein (N); a phosphoprotein (P); a matrix protein (M); a glycoprotein (G); and a ‘large’ RNA-dependent, RNA polymerase (L). Both transcription and replication occur in the host cell cytoplasm (usually, but not exclusively, in a neuron). The viral polypeptides have multiple roles involved in basic structure and key aspects in the viral cycle, from initial cellular reception to transcription and translation, through virion assembly, and viral egress [21,22,23,24,25,26]. Through long-term adaptation to vertebrate hosts, lyssaviruses evolved dual stealth and suppression mechanisms to preserve neural pathways, inhibit apoptosis, and limit host inflammatory responses, including modulation of the interferon pathway [27]. Ongoing global research provides key insights into the elegant mechanisms these pathogens exploit in vivo to perpetuate within their essential niche of the mammalian CNS over space and time.

## 3. History of ‘Rabies-Related Viruses’

For thousands of years, multiple cultures sensed a correlation between the bite of an animal and an ensuing illness resulting in death [28]. ‘Rabies’ (i.e., to ‘rage’ or do violence, in animals) or ‘hydrophobia’ (i.e., applied to *Homo sapiens*) was a common name for this recognizable phenomenon, even if not all agreed as to ultimate causation. Such a ‘transmissible toxin’ may have even been an underlying rationale for the generic definition of the term ‘virus’, broadly interpreted as a ‘slimy liquid’ or ‘poison’. Over the centuries, with evolving scientific philosophies and gradual technical improvements in microscopy and laboratory methods, the seemingly larger agents, such as bacteria and fungi, became differentiable from ‘viruses’, the so-called non-filterable microbes ‘of infinite smallness’ (or by the end of the 19th century, as considered classically by Beijerinck as ‘contagium vivum fluidum’). Visualization by electron microscopy of the particulate nature of the causative agents of tobacco mosaic disease (*Tobamovirus tabaci*), foot-and-mouth disease (*Aphthovirus vesiculae*), rabies (*Lyssavirus rabies*), and many others occurred by the mid-20th century. The fates, celestial events, miasma, spontaneous generation, and innumerable other explanations were superseded by the blossoming science of virology.

**Figure 2:**
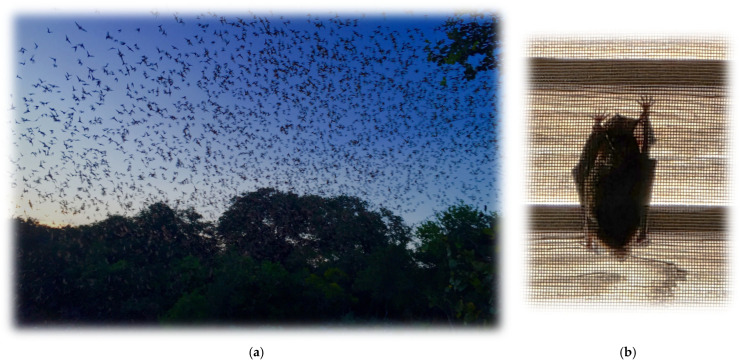
(**a**) Ideal host characteristics, such as high vagility, extreme biodiversity, broad distribution, social gregariousness, and several other attributes intrinsic to bats were increasingly appreciated during the 20th century in the consideration of potential viral reservoirs in the realm of pathogen perpetuation. (**b**) Close-up photo of a single vespertilionid bat during torpor in the attic of a house (Courtesy ME. Rupprecht, Dacula, GA, USA).

Initially, the belief was that only rabies virus (RABV) caused rabies. However, this idea shifted by the mid-20th century, when bats were being recognized as potential viral reservoirs (Figure 2a,b). A comingling of field and laboratory methods broke the monopoly held by RABV with the recognition that closely related viruses could cause the same disease.

**Figure 3:**
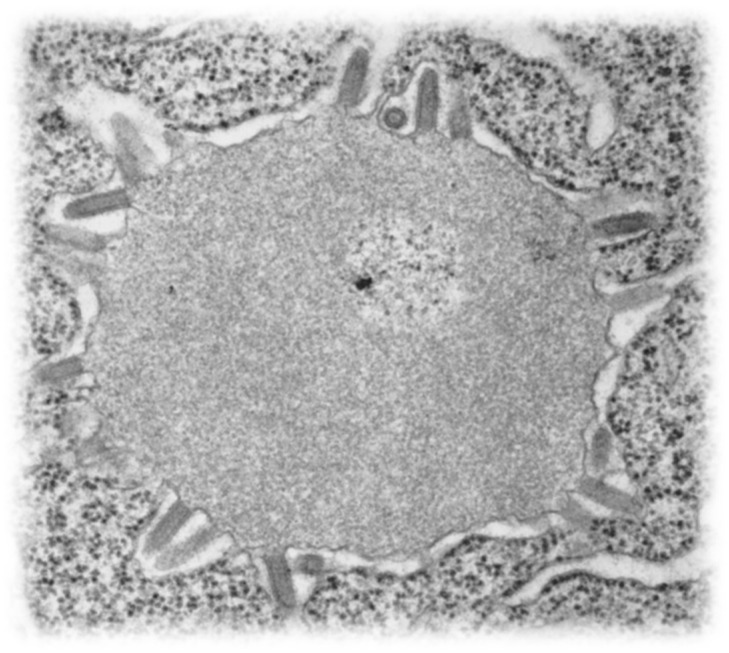
Transmission electron microscopic image illustrating budding, bullet-shaped particles of LBV virions, peripherally surrounding a central, intracytoplasmic inclusion body (Courtesy of F. Murphy, S. Whitfield, US HHS Public Health Image Library).

Although historical accounts from the New World suggested the involvement of vampire bats (*Desmodus rotundus*) since the time of European colonization, documentation for any role of the Chiroptera in viral zoonoses or emerging infectious diseases (EID) awaited the advent of improved diagnostic methods. During the 1950s, field investigations began searching for various agents among different animals for involvement in encephalitides. These global studies involved collections of tissues and viral isolation attempts. One of these ‘virus hunting’ expeditions occurred on Lagos Island, Nigeria, including opportunistic sampling of fruit bats [29]. A brain homogenate from these bats (*Eidolon helvum*) was lethal after intracerebral inoculation in mice. The isolated agent, called Lagos bat virus (LBV), appeared bullet-shaped by electron microscopy, similar to RABV virions (Figure 3).

During the 1960s, another isolate, obtained from Nigerian shrews, also killed laboratory mice [30]. The name given to this isolate was Mokola virus (MOK), a suspected zoonotic agent [31]. Both LBV and MOK appeared similar in morphology to RABV, but distinguishable by serology, designated as Rabies Serotypes 2 and 3, respectively [32]. During experimental infections, RABV-vaccinated mice resisted challenge against MOK only to a minimal degree [33]. Both LBV and MOK produced fatal infections when inoculated into dogs and non-human primates [34]. Comparing RABV, LBV, and MOK, the viral ribonucleoprotein (RNP) appeared as a unifying ‘rabies group-specific antigen’, with the envelope-associated antigen (the G protein) proposed as a serotypic determinant among members [35].

The list of lyssaviruses grew with additional epizootiological introspection. In South Africa, a case of ‘human rabies’ occurred in a man bitten on the lip by a bat, but the immunofluorescent reactivity appeared atypical [36]. Duvenhage virus (DUVV) was the name assigned to this isolate, after the name of the patient who died from rabies [37]. Considering virion morphology, reactogenicity to hyperimmune sera, and the associated encephalitic infection, DUVV joined the ‘rabies serogroup’, as serotype 4.

In 1978, investigators at the Wistar Institute in Philadelphia, PA, USA, reported the generation of hybridomas that produced monoclonal antibodies (MAbs) against RABV and related viruses [38]. These MAbs recognized antigenic variants of RABV and distinguished them from DUVV, LBV, and MOK. Panels of MAbs directed against the viral N and G proteins allowed antigenic typing of isolates from humans, domestic animals, and wildlife [39,40]. While etiological intra-group distinctions were obvious, the different viruses obtained spatio-temporally from various hosts caused the same fatal disease, naturally or experimentally. That is, after viral inoculation, laboratory animals developed an acute progressive neurological disease, a mild-moderate degree of inflammatory infiltrates in the brain parenchyma, intracytoplasmic inclusion bodies, and by electron microscopy, typical rhabdovirus particles were observed budding upon neuronal endoplasmic reticulum and plasma membranes.

From 1968 to 1985, additional viral isolates occurred from European bats and from a bat biologist in Finland, who developed neurological symptoms 7 weeks after a bat (*Myotis daubentoni*) bite, dying 23 days later [41,42,43,44,45,46]. Initially, based on MAb typing, these isolates were considered as a geographic European subgroup (proposed as serotype 5) related to African DUVV [45]. Over three decades, the terminology of ‘rabies-related viruses’ originated from applied research, based on clinical disease, pathologic findings, morphological appearance, serological cross-neutralization, and antigenic differentiation [47]. Gradually, the realization dawned that rabies was not caused by RABV alone [48]. The appreciable benefits gained from the later use of modern phylogenetic methods from the 1980s to date provided further evidence that rabies was due to infection by different lyssaviruses perpetuated within diverse mammalian populations at a global level (Table 1).

Currently, viral taxonomists recognize >20 putative lyssavirus species. More are expected as pathogen discovery and scientific curiosity proceeds. Within lyssaviruses, species demarcation criteria based on genetic differences are deemed somewhat shorter than for some other viral genera. Essentially, this means that at one time, all lyssaviruses might simply have been called RABV. Splitting on phylogenetic grounds alone never implied such viral species differed in the disease they produced in their respective hosts or public health concerns posed as zoonoses. Confusion is understandable among certain biomedical professionals, who may not perceive this fundamental point. Yet, misunderstanding is rife in the literature today, when one reads that ‘rabies is caused by RABV’, despite conflicting data evident since the mid-20th century. Such taxonomic biodiversity is commonplace and expected among RNA viruses, lacking proofreading enzymes: some mutations result in viral extinctions; many variations are neutral; others provide selective opportunities for adaptation and disease emergence. This is the crux for introducing the history of applied nomenclature assigned to the lyssaviruses, within the greater realm of disease appreciation, applicable to veterinary medicine, public health, and conservation biology.

As the bard once raised rhetorically, ‘…what is in a name…?’. In cogitating about disease nomenclature going forward, obviously one should avoid patient names (e.g., ‘Duvenhage’). Similarly, there is minimal benefit gained for the continuation of ‘place names’ (e.g., ‘Lagos bat virus’). Such usage creates stigmatization, bias, and confusion. Also, with the realization of expansive globalization, climate change, host jumps, pathogen translocation, etc., such narrow geographic assignments become meaningless, as diseases expand (or are realized to be far more expansive than believed). Enlisting a fanciful term like ‘Newville Pitbull Xmas Bat Virus Disease’ for a fictitious malady reported initially within the confines of a small town with a seasonal isolate from a single breed of puppy after consuming a bat 3 months previously, becomes limited, superfluous, and misleading, when a novel zoonosis later erupts later throughout the entire region, affecting dogs, cats, livestock, and poultry (and actually unassociated with bats). To avoid misinformation, desirable alternatives should be simple, accurate, communicative, and more relevant to obvious clinical terminology [110].

Inherent to the microbiological field, different parasitic, fungal, protozoal, bacterial, and viral taxa are associated with their same respective, descriptive disease (Supplementary Table S1). Hence, the relationship between ‘lyssaviruses’ (i.e., etiology) and ‘rabies’ (i.e., disease) is no different—rabies is a viral disease caused by RNA viruses in the Genus Lyssavirus. Therefore, the South African man whose name was coined after he succumbed from a bat bite did not perish from the ‘African Duvenhage bat virus’, but rather, he died from rabies (as Merdith et al. 1971 intimated in the original case report title) [36].

## 4. Host/Pathogen and Clinical Spectrum

All lyssaviruses cause an acute, progressive encephalitis. The clinical signs are highly variable but essentially indistinguishable, regardless of viral species. All warm-blooded vertebrates can support a productive viral infection [102]. Birds are susceptible, but reports are rare, with no recent comparative surveys [116]. Among mammals, wildlife reservoirs, responsible for intraspecific perpetuation and interspecific spillover infections, reside within the Chiroptera, Carnivora, and Primates [117]. Other mammalian species serve as vectors or victims. In domestic mammals, the dog is unique, as the sole representative of lyssavirus reservoir, vector, and victim. Felids are effective vectors, but not reservoirs, and are increasingly important in the epidemiology of the disease [118]. Today, unsupervised community dogs in lower- and middle-income countries (LMICs) form the most important public health problems associated with rabies.

Curiously, reports of infection span the breadth of domestic species, from companion animals to livestock, and captive exotic taxa, ranging in extremes from small pocket pets to elephants (Supplementary Table S2). This diversity impacts the traditional practitioner, the zoo veterinarian, and those specializing in the health of managed wildlife [119]. If related case reports for mammalian species are minimal (e.g., marsupials) or lacking (e.g., cetaceans) to date, this may suggest an underlying biological/ecological aspect or reflect a lack of epidemiological introspection. The clinical spectrum of rabies among described taxa ranges from the non-specific, during the prodromal phase, to the dramatic in the acute neurological stages (i.e., so-called ‘furious or paralytic’ manifestations), followed by coma and death, predicated in part by dose, route, severity, species, and the discerning professional abilities via keen observation (Supplementary Table S3).

Understandably, the clinical signs associated with lyssavirus infections are quite variable. While the image of mania conjured from a rabid dog excites the media and public imagination, the onset is non-specific and the paralytic form is much more subtle. Additionally, cases in livestock are frequently overlooked [175]. Nevertheless, some signs, such as altered vocalizations like incessant bovid bellowing, are nearly pathognomonic and once heard, difficult to forget. Moreover, encephalitis in large-bodied animals is a dangerous combination. People have been injured severely or killed outright by rabid cattle and equids.

**Figure 4:**
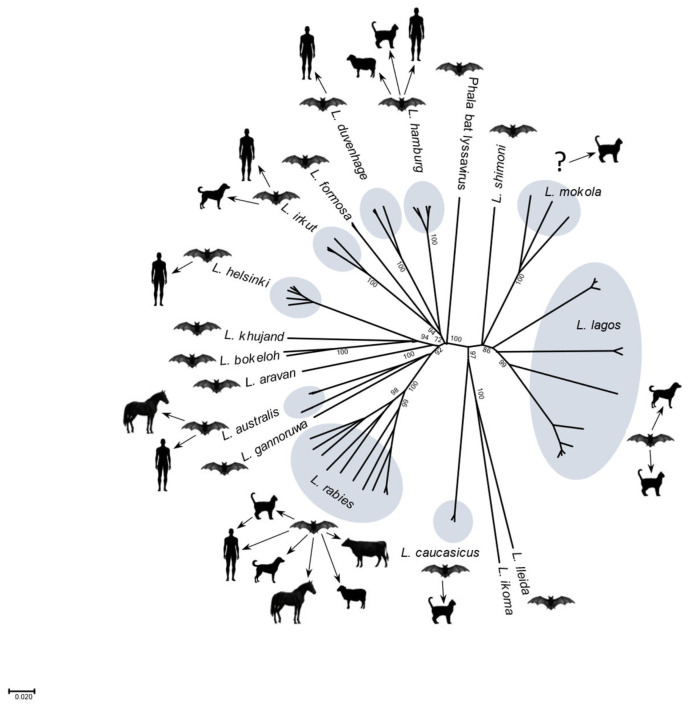
A phylogram of selected lyssavirus species, based upon known or presumed mammalian reservoirs and indications of spillover infections to other species (Courtesy L. Kuzmin, University of Texas Medical Branch, Galveston, TX, USA).

Although RABV is the agent most commonly associated with rabies cases in livestock (especially in Latin America due to depredation from infected vampire bats), other lyssaviruses have also been found in domestic animals. For example, EBLV and WCBV were reported in European cats. Both MOK and LBV were observed in vaccinated African cats and dogs. In Europe, between 1998 and 2002, EBLV1 was identified in five Danish sheep with neurological disorders. In two of the herds from which three diseased animals originated, EBLV1-neutralizing antibodies were detected in one of 69 sheep. In 2000, >2000 sheep sera collected at slaughterhouses were all negative for EBLV1 antibodies, and EBLV1 was not found in 87 animals displaying neurological signs. While other lyssaviruses besides RABV do infect livestock, such as sheep, under natural conditions, such occurrences in Europe appear as incidental events only [76,103,176]. Most lyssaviruses propagate intraspecifically, with unpredictable opportunities for spillover infections to humans, domestic animals, and wildlife (Figure 4). Single viral species isolations with long intervening periods without additional reports imply inadequate surveillance or the possibility of viral extinction.

## 5. Pathobiology

In brief, lyssaviruses enter host peripheral tissues, replicate in the CNS, and are excreted in the saliva [173]. Viral exposure is defined as direct transdermal or mucosal contact with known or potentially infectious material, such as saliva, salivary glands, brain, or related neural tissue [177,178]. Despite multiple routes of potential non-bite exposure, almost all rabies cases are caused by a bite. Rabid animals excrete large numbers of virions and produce copious amounts of saliva. Non-bite exposures include scratches, inhalation of aerosols, contamination of an open lesion, or mucosal contact with infectious materials, such as a lick from a rabid mammal excreting virus in its saliva to a fresh wound, or in direct contact with the nose, mouth, eyes, etc. Following exposure, incubation periods vary from days to months (and in rare cases > 1 year). Length is predicated in part upon the viral dose and route. A myriad of clinical signs are described, none of which are pathognomonic, beyond the notation of uncharacteristic behavior from the norm (Supplementary Table S3, Supplementary Figure S1). In some situations, acute death may occur without any signs observed, whereas in other cases, a morbidity period of 3–4 days may present with either furious or paralytic manifestations [152,179,180,181]. In dogs, cats, and ferrets, viral excretion may not occur, can be concomitant with clinical signs, or be shed 7–10 days before illness, based on naturally infected and experimental studies [165,177,182,183]. Comparative data from other species are lacking. Although some cases of rabies may appear quite dramatic, others are more subtle, and observation alone is inadequate for confirmation, because the differential diagnosis for suspect encephalitis is broad, including other relevant infectious etiologies, toxicity, and trauma.

On rare occasions, lyssaviruses can induce major structural changes during a productive infection of the CNS [173,181]. However, in contrast to the high case fatality and often dramatic clinical signs, pathological changes are usually minimal when examined microscopically. Cases may be easily missed by the veterinary pathologist when rabies is not on the differential diagnostic list. Gross lesions are inconspicuous, but may include evidence of self-trauma, aspiration pneumonia, meningeal hyperemia, or hemorrhages in the spinal cord. Historically, within the CNS, microscopic perivascular infiltrates, or ‘Babes nodules’, and Van Gehutchten’s descriptions of degenerative ganglion cellular changes were noted in some cases but were deemed non-specific and not universal (Supplementary Figure S2). At the beginning of the 20th century, Adelchi Negri, an Italian pathologist, detected oval or spherical inclusions in brain smears of rabid animals, believing them to be indicative of a parasitic microorganism, such as a protozoan [185]. Concomitantly, an American pathologist, Anna Wessels Williams, observed similar microscopic observations [186]. However, Negri published his results first. Hence, the structures were named after him. These intracytoplasmic inclusions or ‘Negri bodies’ (rather than ‘Williams bodies’) may be found in infected neurons, fluctuate in size and number, or may not occur (Supplementary Figure S3).

**Figure 5:**
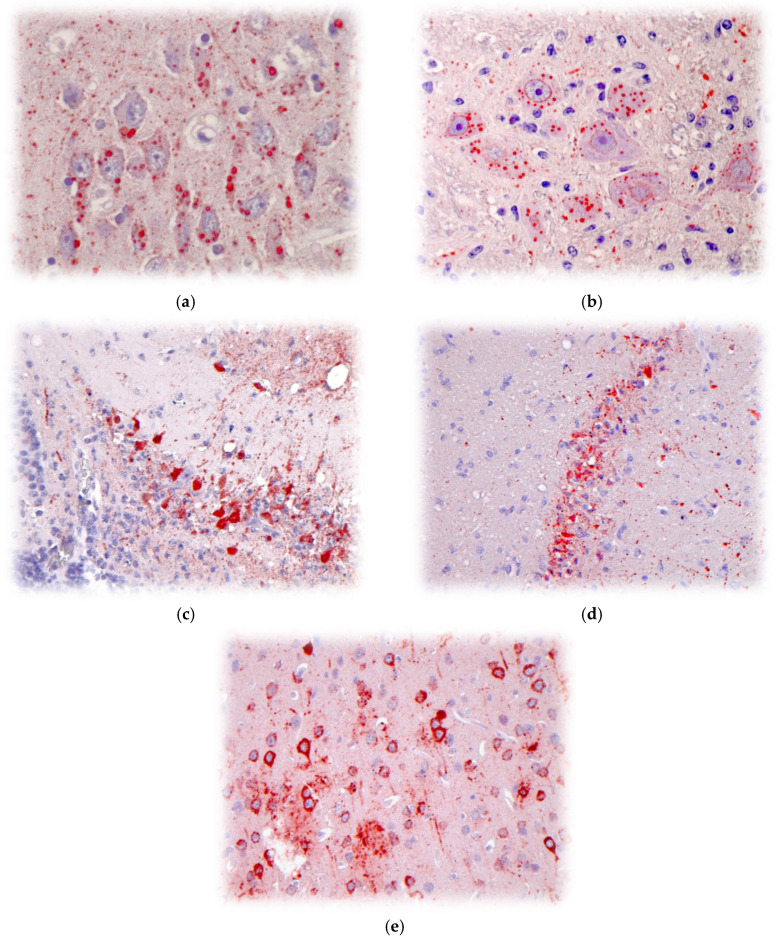
(**a**) Abundant RABV antigens (magenta-stained inclusions) detected microscopically in the brain tissue of a rabid dog by IHC, 400× (Courtesy M. Niezgoda, US CDC, Atlanta, GA, USA). (**b**) Abundant RABV antigens (magenta-stained inclusions) detected microscopically in the brain tissue of a rabid sheep by IHC, 400× (Courtesy M. Niezgoda, US CDC, Atlanta, GA, USA). (**c**) Demonstration of abundant EBLV1 antigens (magenta-stained inclusions) in brain tissue as detected by IHC, after experimental rodent infection, 200× (Courtesy M. Niezgoda, US CDC, Atlanta, GA, USA). (**d**) Demonstration of abundant DUVV antigens (magenta-stained inclusions) in brain tissue as detected microscopically by IHC, after experimental rodent infection, 200× (Courtesy M. Niezgoda, US CDC, Atlanta, GA, USA). (**e**) Demonstration of abundant WCBV antigens (magenta-stained inclusions) in brain tissue as detected microscopically by IHC, after experimental rodent infection, 200× (Courtesy M. Niezgoda, US CDC, Atlanta, GA, USA).

Despite their varying biogeography and antigenic/genetic diversity, the clinical-pathological spectra associated with lyssaviruses are indistinguishable, both in their natural hosts or demonstrable during experimental infection (e.g., RABV, EBLV, DUVV, WCBV), as observed using immunohistochemistry (IHC) or similar techniques (Figure 5a–e) [187]. By comparing lyssavirus species, isolate sources, exposure routes, viral doses, and animal models, a diversity of signs, incubation periods, shedding, survival, immune response, etc. may be observed experimentally, suggestive of different pathogenicity indices, extrapolated to natural hosts [188,189].

Besides histological evidence of mononuclear perivascular cuffing, glial nodules (with limited neuronal degeneration), neuronophagia, and negrigenesis, other pathological changes in the CNS of domestic animals may include spongiform changes in the neuropil of the grey matter [173,190,191,192]. As histopathological lesions may be absent, minimal, or missed during neuronal dysfunction, specific diagnostic tests are required to confirm clinical suspicions of lyssavirus infection.

## 6. Laboratory Diagnosis of Lyssaviruses in Animals

Reliable detection of lyssaviruses relies upon a network of diagnostic tests that can identify and confirm cases of rabies. Typically, animals are tested for the presence of lyssavirus infection through two major paths: subjects identified as threats to human or animal health (usually via passive surveillance); and wildlife monitoring (often via enhanced surveillance). Diagnosis is reasonably straightforward in an abnormal suspect with compatible signs, known to have been exposed over the past several weeks to months to a laboratory-confirmed rabid animal (e.g., a now emaciated, paralyzed, unvaccinated outdoor cat that escaped but reappears at the ‘owner’s’ premises, 6 weeks after catching a sick bat that tested positive). Unfortunately, things are rarely simple, as the prodromal onset is nonspecific, and many exposures may go unrecognized, particularly in free-ranging animals. In humans, antemortem methods, performed at national reference centers, may be utilized in patients with suspected viral encephalitis [177]. In other animals, postmortem techniques are the norm, for a variety of reasons. Notably, rabid animals pose a significant public health hazard to owners and veterinary staff. Considering medical options, aggressive behaviors, self-mutilation, end-stage suffering, etc., provides a logical rationale for euthanasia by a veterinarian, as the morbidity period can be short and extreme, recognizing the disease is not treatable. Importantly, if an animal has exposed a person, speed is of the essence for notification, as part of the risk assessment for human postexposure prophylaxis (PEP), which includes wound care, vaccination, and for bites to the naïve patient, rabies immune globulin (RIG)/MAbs [177,178]. In addition, local animal health regulations may preclude the application of certain types of intra-vitam testing (except for the assessment of immune response to vaccination or serological monitoring for epizootiological insights).

Over the past 75 years, a variety of laboratory techniques evolved for an appropriate postmortem diagnosis of rabies in animals, focused on the detection of lyssavirus antigens, nucleic acids, and antibodies [193,194]. Such tests are sensitive, specific, rapid, and economical, dependent upon fit-for-purpose needs [195,196,197,198,199,200,201,202,203,204,205,206,207,208,209,210,211,212,213,214,215]. Additionally, these tests can be used in conjunction with one another as ‘screens’ to reduce the overall costs of detection. If histology suggests lyssavirus infection, specific laboratory testing can confirm initial suspicions. Pathology tissue blocks provide opportunities via IHC if fresh/frozen samples are unavailable (Figure 5). Other classical methods, such as electron microscopy (Figure 3), viral isolation, etc. may be useful for applied research purposes, but are rarely employed today for routine diagnostic applications [193,194].

A primary consideration in laboratory detection of lyssaviruses should be the sample types collected for testing. Since lyssaviruses are neurotropic and illness onset begins after viral replication in the CNS, a full cross-section of the brainstem (and all three lobes of the cerebellum) is the most reliable sample for diagnosis and is required for a definitive rule-out of infection [202,216]. Other portions of the CNS may be negative while the brainstem tests positive. Antemortem testing of different tissue types (e.g., skin biopsy, saliva, csf, sera, etc.) is possible in theory, but such methods are typically reserved for suspect rabies cases in humans. The tissues required for antemortem testing are invasive to the patient and may not rule out rabies due to the difficulty in accurately collecting and testing these materials in a timely manner as well as a possible lack of analyte in non-neuronal tissues. In all cases, proper collection, storage, and rapid transportation of samples are crucial to preserve the integrity of the analytes targeted by different test methods [217]. Once samples arrive at a laboratory, it is imperative that samples are handled carefully, and that all sources of cross contamination are eliminated.

**Figure 6:**
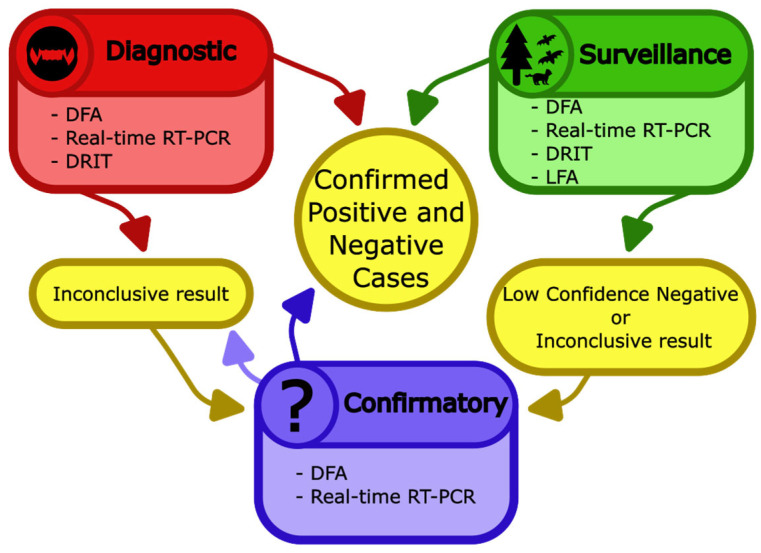
Laboratory and field tests for detection of lyssaviruses in animals.

Results from diagnostic and surveillance tests can confirm lyssavirus infections, sometimes rule out lyssavirus infection, or lead to inconclusive results. Inconclusive results and low confidence negatives (for instance, negative results from lateral flow assays, LFAs), should be sent to a reference laboratory to undergo confirmatory testing. This secondary testing may resolve or uphold the inconclusive result, depending on the condition and type of sample (Figure 6).

### 6.1. Nucleic Acid-Based Assays

Nucleic acid-based assays are designed to extract and amplify target RNA within a sample. One major advantage of nucleic acid-based assays is the flexibility to specifically target broad and narrow representatives of the *Lyssavirus* genus. Laboratory-developed tests range in scope from detecting sub-variants of RABV to detecting all known lyssavirus species [199,200,218,219].

Conventional RT-PCR was the first of these types of tests. In short, extracted RNA is amplified to make cDNA, which is then visualized in a gel via electrophoresis [193,195,196]. Later, this method would develop into hemi-nested RT-PCR, where the cDNA amplicon from RT-PCR is used as template DNA for an additional PCR reaction [197]. The hemi-nested approach is more sensitive and has been used for decades to confirm rabies cases around the world. However, it is also more susceptible to false positive results because contaminant cDNA can be amplified in the secondary reaction.

Recently, advancements in PCR technology have led to the development and improvement of two real-time RT-PCR pan-lyssavirus diagnostic tests: JW12 and LN34 [193,198,199,200,201]. These tests improve upon the sensitivity of conventional RT-PCR assays by adding probes tagged with fluorescent dyes that emit fluorescence upon replication of target RNA. Additionally, real-time RT-PCR can be completed in single-well, one-step reactions, which reduces the potential for cross-contamination. Both tests have been validated thoroughly for their use in human antemortem and animal postmortem diagnostic testing and serve as tools to screen samples or confirm the results of another diagnostic test (e.g., direct fluorescent antibody test, DFA; direct rapid immunohistochemical test, DRIT; etc.).

Additional advancements in nucleic-acid-based assays have given rise to isothermal amplification methods, which may be more accessible in resource-limited environments or in the field because they do not require thermal cyclers to complete the amplification reaction. Loop-mediated isothermal amplification (LAMP) and Recombinase Polymerase Amplification (RPA) assays have been developed to detect RABV. However, LAMP and RPA assays often do not detect all RABV variants and none have been developed to detect other lyssaviruses [533,534,535,536,537]. New isothermal amplification assays should be validated before employing these methods for diagnostic or surveillance use to ensure sensitivity to all local lyssaviruses.

As a diagnostic tool, nucleic acid-based assays are effective across the lyssavirus space, especially in scenarios where other analytes are degraded or limited [217,218,219,220,221,222]. These assays have also been used to detect and characterize novel lyssaviruses, which may not react predictably in other diagnostic tests [67,86,94]. Lastly, nucleic acid-based tests serve as confirmatory tests for other methods to resolve inconclusive results [198,223,224,225]. In comparative studies, well-developed RT-PCR assays show similar or improved sensitivity and specificity to DFA [198,225,226,227]. Like all diagnostic assays, nucleic acid-based tests should be re-evaluated periodically to ensure proper coverage of target species and viral variants. Additionally, further improvements in these assays make them more efficient and accessible to laboratories by streamlining protocols and reducing costs.

### 6.2. Antigen-Based Assays

**Figure 7:**
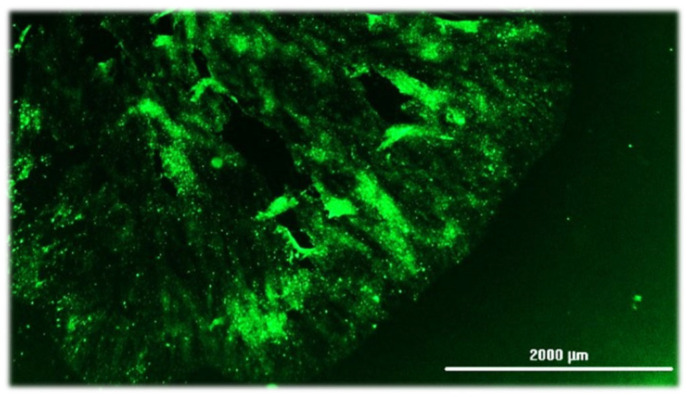
Positive result of a direct fluorescent antibody test performed upon a brain impression of a naturally infected rabid cat (Courtesy R. Davis, Kansas State Veterinary Diagnostic Laboratory, Manhattan, KS, USA).

Antigen-based assays have been developed to detect and visualize lyssavirus antigens in tissue samples. This group of tests includes the DFA, which is one of the longest-standing gold-standard diagnostic tests for rabies, due to its high specificity and sensitivity [203]. The core method of antigenic assays is to take representative brain tissue samples, apply antibodies that bind specifically to lyssavirus antigens, and then visualize the antigen-antibody complexes (Figure 7). Antigenic tests rely on the quality of tissue available, the availability of suitable antibodies, and the experience of the technician staff performing the assay [193,202,204]. Studies demonstrate that decomposition reduces reactivity in antigen-based tests, which leads to false negative or inconclusive results [217].

In all antigen-based assays, the antibodies selected must be reactive to all locally circulating lyssaviruses [204,205,227,228]. As such, laboratories must validate any new antibody conjugates to ensure sufficient sensitivity and specificity to local lyssavirus species and variants. Typically, at least two MAb conjugates are used in antigenic testing to ensure coverage for local variants because any singular MAb may not react to all known lyssaviruses and variants of lyssaviruses [10]. Historically, polyclonal antibodies showed broader reactivity to RABV variants compared to MAbs. However, further development and validation are required to ensure the broad reactivity of any analytes to other viral species and variants [229,230,231].

The DRIT was developed at the end of the 20th century and follows a similar protocol to DFA, with a few changes that make the assay rapid and easier to implement. This protocol takes approximately an hour to complete. The use of biotinylated anti-N protein MAbs or polyclonal serum removes the requirement for a fluorescent microscope. Instead, slides of brainstem impressions produced during the DRIT are analyzed with a light microscope [193,206]. Independent validations have shown similar sensitivity and specificity between DRIT and DFA and the ability to detect non-RABV lyssaviruses [221]. The DRIT is used routinely in enhanced surveillance of rabies in wildlife and to diagnose rabies cases. However, as with any diagnostic test, inconclusive results should be further analyzed by a confirmatory test [86,232,233].

In contrast to DFA/DRIT, LFAs are a different method of antigen detection using a brainstem tissue sample, garnering international attention in recent years. The low cost of LFA devices makes this method attractive, though there are lingering concerns about the reliability of these tests to detect the presence of all lyssaviruses. In 2023, the international organizations released a statement on this class of diagnostic devices, cautioning against their use as a primary method to diagnose cases of rabies [234].

Evaluations of LFAs have shown large variations in diagnostic quality depending on brand, lot, and adherence to manufacturer instructions [207,208,209,210,211,235,236,237]. To the consternation of researchers, companies that produce LFAs often do not disclose the specific antibodies that react to antigens present within the sample. As mentioned above, antigenic detection techniques are reliant upon the proper selection of antibodies that react to all locally circulating lyssaviruses. To date, studies have shown conflicting sensitivity against various lyssaviruses [208,209,210,211]. In addition to concerns about the applicability of LFAs beyond RABV to other diverse lyssaviruses, strict adherence to biosafety and the use of personal protective equipment (PPE) is paramount for technicians handling infected CNS tissues. Current LFAs are normally performed in a field environment, without routine laboratory biosafety equipment such as a BSC. As such, technicians should use appropriate PPE and homogenize CNS tissues carefully to avoid aerosols. Technicians should also be aware that preexposure prophylaxis (PrEP) and PEP, if available, may not provide sufficient immunization against lyssaviruses in phylogroups II and III [86,177,238].

While LFAs are not appropriate for ruling out lyssavirus infection, they have become a promising surveillance tool to screen field samples before pursuing further evaluation. In this capacity, many regions have implemented LFAs to detect positive cases while routing potential negative samples to more robust diagnostic methods [236,237,239]. More research is needed to determine if certain brands of LFAs can be useful to screen for lyssavirus species besides RABV due to the limitations of the antibodies used to produce these devices. In addition, they should not be used for attempted detection in saliva or other unsuitable sample types, for both pathobiological and testing validation concerns.

### 6.3. Serum-Based Assays

Serological assays are not generally used for veterinary diagnostic testing per se, because sufficient antibodies are usually only present (if at all) in very late progression of rabies [240]. Animals usually die before current diagnostic methods can identify positives because of late seroconversion and individual immune responses by different species, to various lyssaviruses, exposure routes and doses, etc. In humans, serological assays are used in antemortem human testing to evaluate immune responses in CSF and serum. Combined with other assays, such as real-time RT-PCR and DFA, serological assays, such as the rapid fluorescent focus inhibition test (RFFIT) or fluorescent antibody virus neutralization test (FAVN), contribute to a robust testing algorithm that assesses lyssavirus activity in antemortem (or postmortem) samples.

The detection of VNA in assays such as FAVN and RFFIT uses dilutions of patient serum or CSF (and positive/negative control antibodies) to inactivate standard doses of replication-competent lyssaviruses in the test. Naive cells are added to the sample/virus mixture, where any remaining, non-neutralized virus infects and replicates over a defined period. Infected cells are fixed and conjugated antibodies are used to visualize viral antigens (as in the DFA). Each dilution is scored to determine the end-point titer of the patient, compared to standard positive and negative controls [212,213]. The RFFIT/FAVN approach is effective in assessing the immune response to vaccination in patients, aids in antemortem diagnostic testing, and can provide useful surveillance data in animal populations. However, it is not recommended as a primary veterinary diagnostic test. Additionally, the antibody conjugates used in this method require similar considerations to antigen-based assays. In contrast to neutralization-based tests, few ELISA or ELISA-like assays exist to detect and diagnose the multiplicity of lyssaviruses [214,215]. These types of assays may prove to be a simpler alternative to detection of antibodies in the future. However, further validation is required before such assays are used routinely.

As geographic regions move toward the goal of rabies control, prevention, and selective elimination of RABV variants (i.e., canine), the need for enhanced surveillance and the ongoing capacity to identify new rabies cases does not disappear. The diagnostic methods outlined above function best for the detection of RABV specifically. Many methods have mixed results when applied to other lyssaviruses. This is not surprising as RABV is the most ‘successful’ member of the Genus and has the greatest health impact globally. However, enhanced capacity to detect and prevent lyssavirus transmission will be critical as programs work towards the goal of canine rabies elimination. Further research in this field could yield more robust and efficient assays for the routine detection of all lyssaviruses as well as broaden acceptable sample types for surveillance purposes. Finally, impacts from neighboring areas and conflict zones will remain an ongoing threat in many regions. As such, robust diagnostic networks will be required to halt the spread of rabies and prevent ongoing viral transmission among a diversity of organismal and viral species, where feasible.

## 7. Lyssavirus Occurrence and Disease Designations

The burden of animal rabies in both highly developed and LMICs is often unknown. Reports may not be notifiable, registering significant chagrin among stakeholders, policymakers, and supporters [241]. Cases based on clinical signs alone are gross underestimates of true incidence. Surveillance may be focused more on companion pets in urban areas, but less so on production animals in rural communities. For example, in a developed country that eliminated canine rabies but in which wildlife rabies perpetuates, such as the US, the estimated incidence may be small, less than 0.1 cases per 100,000 head of animals tested per year [242]. In contrast, within a country such as Türkiye, with both dog and wildlife rabies, rabies cases in livestock may vary annually from approximately 0.1 to 3.9 cases per 100,000 head [243]. In Ethiopia, the estimated incidence of canine rabies was 413 cases per 100,000 dogs, with a high economic impact on pastoral cattle production [244,245]. In another region of the country, 44.9 human cases per 100,000 population and 3.4 animal cases per 100,000 population were registered from 2017 to 2021, showing an obvious and not infrequent disconnect between public health and agricultural sector reporting [246].

Surveillance bias is obvious by species and regions. Outbreaks from canine rabies may impact several species of livestock, as observed from rural areas throughout Asia, as in China [247]. In regions where both canine and wildlife rabies perpetuate in concert with semi-nomadic cultures, cases are widespread. Compared to cattle, reports of rabies in swine are infrequent in general, although local outbreaks have occurred on occasion [143]. In Oman, where sylvatic rabies predominates, goats and camels were the most frequently reported rabid animals during 2017–2019 [248]. Since an initial reported case in Sudan in 1961, rabies in equids has been documented consistently [249]. Despite underreporting, whether the enzootic cycle involves rabid dogs (e.g., Indian subcontinent) or wildlife reservoirs (e.g., South America), spillover infections to livestock are not uncommon, causing major hardship to the local farming community.

In areas where canine rabies has been eliminated and rabies among wild carnivores controlled via oral vaccination, reports of rabies overall may be quite rare, such as occasional cases of spillover via rabid insectivorous bats (e.g., Western Europe). As a major exception, within the Americas, from Mexico to Argentina, outbreaks of bovine paralytic rabies cases may number into the tens of thousands annually, secondary to infection by hematophagous vampire bats [250,251]. One study in Brazil estimated more than 30,000 cases of infected cattle per year [252]. In contrast, from 1999 to December 2022, only 50,944 total cases of rabies in herbivores were recorded nationwide [253]. Often, even a single case exemplifies an outbreak, as ranches may be far from diagnostic facilities, making sample collection and shipment onerous [254]. Depending upon climate change predictions, vampire bat expansion (and the threat of rabies) may include broader parts of Argentina, Chile, and the US [255].

Beyond the dynamic realm of pathogens, hosts, and environments, there is the biopolitical reality of independent philosophies of each country related to self-reporting as to disease definition, outside the academic fields of virology, epidemiology, and taxonomy. In actuality, very few localities are absolutely ‘free’, meaning no lyssavirus detection with an adequate, ongoing laboratory-based surveillance system and disease notifiabilty. Under an enlightened scheme, one could question the apparent absence of rabies in Antarctica, without any diagnostic denominator data over time, given the diverse and abundant marine mammals there and other insular locations throughout the Southern Ocean. Similar questions could be raised about Oceania.

Obviously, the elimination of canine rabies is a major achievable global milestone. Countries go to great expense to acquire such status and prevent re-introduction [256]. Criteria for various aspects of ‘freedom’ vary among international health organizations [257]. For example, the WOAH sets standards for international trade in terrestrial animals. They define rabies as an infection caused by RABV, irrespective of animal species. A ‘rabies-free’ country has no indigenously acquired RABV case in the previous two years. Most of the global burden is due to RABV, with a broad host spectrum reported among mammals, as an exception to other lyssaviruses [103]. The WHO is somewhat similar but has different health risks predicated upon the absence of all lyssaviruses, wildlife rabies (excluding bats), or dogs. Thus, different localities may or may not be considered to have rabies, depending on the selected classification scheme (Table 2). Paradoxically, this means that laboratory-confirmed human, domestic animal, or wildlife deaths from lyssavirus infections would not necessarily affect the rabies status of a locality.

Primary vaccination with licensed products (i.e., PrEP) would reasonably minimize the risk of viral infection for most domestic animals, at least for Phylogroup I lyssaviruses. However, this strategy for prevention and control needs to occur on a routine basis, without the actual prospect of fundamental ‘eradication’ (as achieved for smallpox or rinderpest), because of the reality of disease maintenance among bat reservoirs and lack of biologics with efficacy against Phylogroup II and III lyssaviruses [238,258].

## 8. Management

The management of animal rabies spans the gamut from the individual (e.g., companion animal), to variable-sized groups (e.g., herd health), and wide-ranging populations (e.g., wildlife vaccination programs) [152,177,259,260,261,262,263,264,265,266,267,268,269,270,271,272,273,274,275,276,277]. While the concept of control includes elements of diagnosis, euthanasia, movement restrictions, quarantine, etc., perhaps the most significant managerial aspect lies in prevention via proper animal PrEP within an integrated national program [152,157,278,279,280,281,282,283,284,285]. Multiple variables affect the response of a rabies vaccine in animals. For example, even in a single species, the probability of success in vaccinations of dogs against RABV may depend on overall health, the type of vaccine used, potency, number of rabies vaccinations, the breed, age at vaccination, and number of days after vaccination when the animal might be exposed [286,287,288,289,290,291,292,293,294,295,296].

Despite many variables, rabies is quite uncommon in properly vaccinated animals. Failures may occur, albeit infrequently, so should be included in the differential diagnosis for any animal with clinical signs compatible with rabies regardless of vaccination history [297,298,299,300]. Continued surveillance is imperative to document such cases and identify epidemiological trends in field programs. Rare vaccine failures are possible even against homologous viruses, but perhaps more so against heterologous lyssaviruses, as found with LBV and MOK in the field and others in the laboratory [92,98,100,238,301,302,303,304]. Attempts towards the development of a panlyssavirus vaccine continue [305,306,307].

Over the past century, animal vaccines improved in purity, potency, safety, duration of immunity, efficacy, and cost. The first veterinary vaccines were crude nervous tissue-derived products. These were replaced by a second generation of MLV. A third generation involved inactivated biologics produced in cell culture, usually containing an adjuvant, such as aluminum hydroxide. The fourth generation encompasses recombinant products. Over the past decade, many different veterinary vaccines have been produced globally for administration to companion animals, livestock, or wildlife, for management against infection by lyssaviruses (Supplementary Table S4). Besides pets and livestock, those captive animals held in public settings, including petting zoos, should be vaccinated as well. As there are no vaccines licensed for all mammals at risk, veterinary approval for off-label use is recommended.

Despite the broad diversity of producers and vaccines, the number of doses required and actually manufactured is insufficient to meet global demand. For example, in China, more than 40 million additional doses are needed from the domestic and imported sectors, just for dogs and cats [267]. Health disparities are obvious in rural areas and viral emergence via wildlife remains a concern in Asia and Africa [270]. The availability of vaccines for several types of domestic livestock, such as camels, is inadequate [308]. Partnerships between animal protection organizations and local governments may provide one solution in tailoring successful veterinary programming for specific rural conditions [269]. In the emergence of future lucrative markets, caution is urged against the illicit use of counterfeit vaccines in veterinary medicine, as seen with human rabies biologics [309,310]. Other concerns arising after the COVID-19 pandemic are associated with vaccine hesitancy, both in developed and LMICs, at a crucial time when the use of modern safe, effective, and free veterinary vaccines is required to protect animals at risk and their owners [311,312,313,314,315]. Besides the consideration to promote vaccines for animals at risk prior to viral exposure, they may be too young or unlisted based on specific product label requirements and may be missed for other reasons. Opportunities for PEP in the naïve animal are also an option in an evidence-based manner, when permissible under local veterinary regulations [316,317].

The continued use of licensed biologics for prevention against a productive lyssavirus infection is dependent in part on the quality of specific vaccines, as well as the life history stage and exposure status of the proposed vaccine, and the experience of the veterinary vaccinator (Table 3). Broad guidance is based on general immunological parameters and best biomedical practices. Given the complex interplay among host species, infectious agents, environmental variables, and available products, engagement with local subject matter experts and government authorities is always recommended for local compliance.

## 9. Selected Regional Epidemiological Highlights

### 9.1. Europe

Despite the significant regional impact of the two World Wars during the 20th century, substantial progress in the diagnosis, control, and prevention of lyssaviruses occurred on the continent over the past 200 years [318]. For example, in the late 19th century, the rabies vaccine concept originated in Europe. After centuries of endemicity, the control of canine rabies began, even before the advent of vaccination, consisting mainly of free-roaming dog culling, leash laws, mandatory muzzling, and improved hygienic measures. Thereafter, parenteral canine vaccination rapidly demonstrated its effectiveness in controlling and eventually eliminating canine rabies.

At the end of the 1970s, Europe was the first continent to utilize oral vaccination of wildlife, paving the way for others to consider [271,319]. This method proved highly effective in controlling the disease in foxes and raccoon dogs across the European Union (EU), which was close to rabies elimination in 2019 [266]. For example, in 2018, only eight RABV cases (six in wildlife and two in domestic animals) were reported in just three EU Member States. This represented a major decrease compared to 2010 when there were more than 1500 cases in nine EU Member States. The goal was to reach zero cases in wild carnivores and domestic animals in the EU by 2020, a target that seemed achievable at that time. Widespread control of wildlife rabies in Western Europe lessened overall agricultural and public health threats. Today, veterinary vaccination in the EU is on a case-by-case basis due to canine rabies elimination. Primary risks are to foreign travelers and the translocation of cases from other areas where dog RABV perpetuates [320,321,322,323].

As elsewhere throughout the world, bat rabies recognition began during the 1950s [324]. To date, besides RABV, at least six other lyssaviruses are found throughout Europe [325]. A total of five enzootic human cases from EBLV were reported, but not from the other indigenous bat lyssaviruses, yet [302]. One human case of DUVV was imported to the Netherlands from Kenya, where the unvaccinated traveler was exposed but unaware of indigenous bat rabies in Africa [64,326].

**Figure 8:**
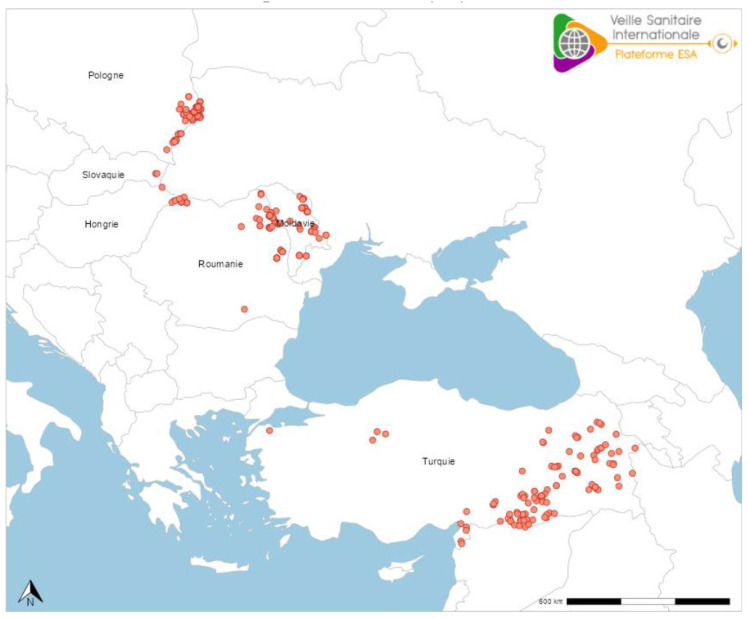
Map showing reported RABV cases (red circles) in parts of Central and Eastern Europe from January 2024 to 12 May 2025 in countries reporting data to the Animal Disease Information System (https://food.ec.europa.eu/animals/animal-diseases/animal-disease-information-system-adis_en, accessed on 19 May 2025). Source of the figure: Epidémiosurveillance Santé Animale Platform (https://www.plateforme-esa.fr/fr, accessed on 19 May 2025).

Conflict zones in Eastern Europe jeopardize the substantial agricultural, public health, and economic progress gained throughout this region [327,328]. Political events in Ukraine impact disease prevention and control (Figure 8). For example, the local rabies situation deteriorated in Moldova, Romania and Poland. Other surrounding countries where rabies in wild mesocarnivores was eliminated (i.e., Slovakia and Hungary) were re-infected recently. The current distribution of RABV in Europe demonstrates the fragility of the epidemiological situation in any locality—closely linked to that of bordering countries. Such prior success created unexpected challenges to maintaining public education about lyssaviruses, rabies vaccine supplies, and focal disease prioritization in a context of other EIDs over the past decade (e.g., African swine fever, foot and mouth disease, highly pathogenic avian influenza, blue tongue, lumpy skin disease, etc.) [2,3,6,329,330,331,332].

### 9.2. Asia and the Indian Sub-Continent

The largest burden of animal and human rabies lies in Asia [333]. Besides RABV, at least six other recognized or putative lyssaviruses belonging to phylogroup I have been documented across the region, from Central Asia, China, and Sri Lanka [51,52,53,60,66,67,69,87,109]. A 2007 lyssavirus isolate from a fatal case in a 20-year-old female, who was exposed to a bat in her village of Ozernoye in the Russian Far East, was originally called Ozernoe virus [334]. Later analysis showed this was IRKV and several other human cases have occurred [87,538]. Very little is known about the broader distribution or epidemiological significance of such non-RABV lyssaviruses, highlighting a significant gap in current surveillance and research efforts.

Reports and models estimate that India, China, and the Philippines are some of the most affected countries in Asia. Within the region, China has demonstrated progress with a gradual decrease in human cases. In 1981, there were reports of >7000 human rabies infections, declining to <1000 by 2014 [335]. Only 202 human cases, located in 143 areas, were recorded in 2020 [336]. Continued utilization of a One Health approach suggests that the country could meet the goal of ‘Zero by Thirty’ (ZBT), although foci in rural areas remain a challenge [270]. Such optimism may not be realistic in other areas [337]. For example, in the Philippines, there has been an increase in reported cases between 2022 and 2023, to >350 human deaths per year [338]. As an archipelago, viral transmission appears impeded by the ocean, with little inter-island transmission, which could be exploited to improve upon future dog rabies control programs [339]. The Indian subcontinent serves as a stark contrast to the steady progress witnessed by China, or the potential barriers provided by the sea in the Philippines. India lacks reliable data on human fatalities or rabid animals, even though laboratory methods have improved substantially [340,341].

Most of the estimated global burden of rabies is believed to occur in India. To date, RABV is the only documented lyssavirus circulating in India, with domestic dogs serving as the primary reservoir and transmission vector [177,284]. A report of a rabid gray-headed flying fox (*Pteropus poliocephalus*) infected with a ‘rhabdovirus’ from northern India appeared in 1980, but with no further characterization [342]. More recent surveillance of during 2013-14 in Nagaland, a northeastern state of India (where regionally each year thousands of bats may be harvested for consumption and use in local traditional medicine), provided serological evidence of bat exposure to some undefined lyssaviruses [343]. However, a similar survey of bats in Assam found no evidence of serological exposure to RABV or any other lyssaviruses [344]. Such epizootiological use of serology may be insightful for lyssavirus activity but is highly dependent upon the laboratory protocol and isolates employed.

India accounts for an estimated 20,000 human deaths from dog-transmitted RABV each year, resulting from approximately 20 million dog bite cases annually [333,345,346]. While most of these fatalities are believed to be from RABV, the impact of other lyssaviruses is unknown because ‘…these infections may easily be missed since the illness is indistinguishable from rabies encephalitis, which is almost always diagnosed clinically in *Africa and Asia.’* [347].

**Figure 9:**
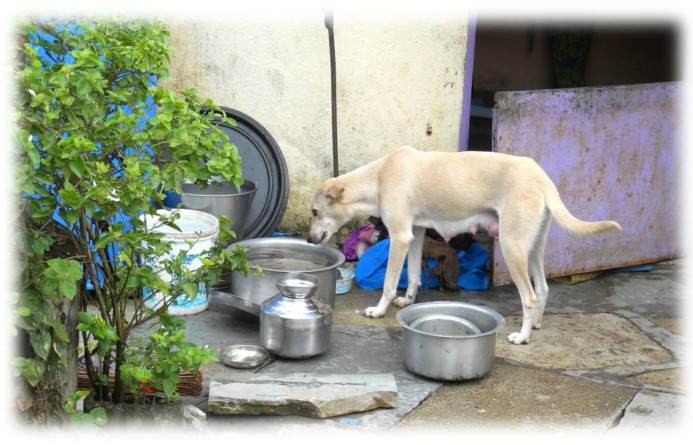
As in many LMICs, free-ranging dogs in India are abundant and dependent upon local community sources for food and shelter.

The burden within India is unsurprising, given the country’s unusually large population of free-roaming dogs, estimated at over 60 million [177,348,349]. These ‘free-roaming’ dogs in such settings are not necessarily “strays” in the conventional sense, because most dogs are loosely affiliated with neighborhoods, making them “community-owned” rather than truly ‘ownerless’ dogs [350]. Moreover, even dogs that are considered ‘owned’ typically roam freely for most of the time, as the concept of dog ownership in these settings does not necessarily extend to routine vaccination, preventive healthcare, birth control, or confinement (Figure 9). The metapopulation dynamics of rabies across large-scale free-roaming and unvaccinated dog populations throughout the Indian subcontinent, coupled with the ongoing, unsupervised interactions of free-roaming dogs with humans, domestic animals, and wildlife, pose major challenges for rabies control programs that rely on dual mass vaccination of dogs and sterilization as a primary means of ‘animal birth control’ (ABC-ARV).

Historically, mass dog vaccination (MDV) programs have been the mainstay of recent successful efforts to control and eliminate canine rabies, thereby reducing human rabies deaths at a regional level [351,352]. For example, countries throughout Latin America and the Caribbean have collectively vaccinated more than 40 million dogs annually against rabies for over three decades [353]. This was achieved because of the strong political commitment of the Pan American Health Organization (PAHO)-coordinated continental scale dog rabies vaccination campaigns [351,353,354]. However, implementing a similar MDV strategy in India without strong political commitment presents significant challenges. Such canine populations have high turnover rates, which is responsible for a continuous reduction in vaccination coverage between annual vaccination campaigns [355]. Moreover, to effectively halt RABV transmission, ideally at least 70% of the regional dog population should be vaccinated within a short (e.g., one month) period [356]. Additionally, controlling RABV outbreaks requires not only MDV but also the euthanasia of suspected rabid animals, along with focal and perifocal vaccination efforts to contain viral spread [351]. However, in India, the policy of dual sterilization and anti-rabies vaccination (i.e., ABC-ARV) established in 2001 has been interpreted in some spheres to prohibit the killing of dogs suspected to be rabid [357]. With an estimated 85 million dogs, over 70% of which are free-roaming, achieving sustained and coordinated vaccination coverage is a commendable but unattainable goal in India within the timestamp of the ZBT.

**Figure 10:**
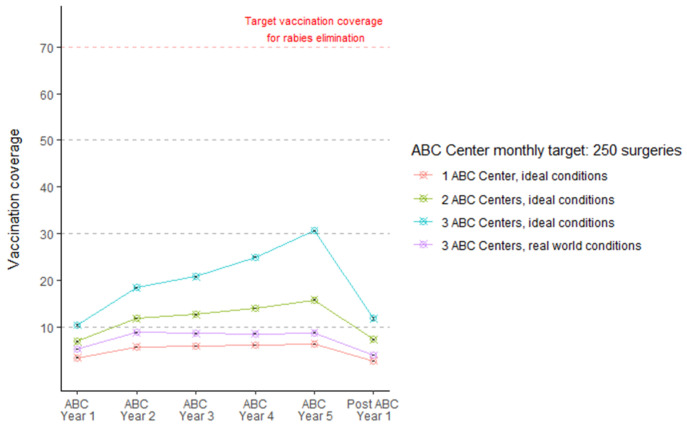
In India, current ABC programs fail to achieve/sustain vaccination coverage necessary for rabies control, even under ideal conditions.

Current ABC-ARV programs aim to reduce dog population turnover and decrease RABV transmission by reducing the number of susceptible dogs and by limiting certain aspects of male dog behaviors, like local dispersal and fighting [358]. In real-world scenarios, these ABC-ARV programs often fail to achieve both the desired reduction in dog population size and the necessary rabies vaccination coverage [359]. For example, using DogPopDY, an agent-based modeling tool that simulates realistic in silico dog populations, even under ideal conditions—such as a closed population with all dogs equally and easily accessible—ABC-ARV programs failed to achieve the vaccination coverage required to interrupt RABV transmission in dog populations (Figure 10). This failure is due primarily to the sheer scale of India’s free-roaming dog population, logistical challenges that hinder regional coverage, and, most critically, a longstanding lack of overall strong political commitment.

As the primary reservoirs and vectors of RABV in India, domestic dogs are also the leading source of viral transmission to other domestic animals. Throughout India, free-ranging cattle are ubiquitous in both urban and rural sites. Dog-mediated spillover to Indian cattle (with more than 25 unique breeds) is not uncommon (e.g., [360,361]), although it is likely under-reported [333,362]. In rural areas, cattle suspected of having rabies, often following known dog bite incidents, are sometimes sold in markets to unsuspecting buyers. This raises further concerns about the perceived risk of viral transmission to humans through non-bite exposures, including the potential for foodborne transmission, albeit rare. As noted historically by Fleming (1872), …’a large share of proof is in favor of the innocuousness of the milk derived from cows or other creatures while affected with rabies…’, yet still safely opining ‘…it must always be remembered that one positive fact is worth a thousand negative ones…’ [184]. While there is no documented evidence confirming the spread of RABV through the consumption of meat or dairy from infected animals, the theoretical risk of transmission in raw (unpasteurized/unboiled) milk from infected cattle raises frequent media attention and public concerns [363]. Anecdotal reports exist, including a recent reported case in India involving a woman who developed rabies after consuming unpasteurized milk [364].

With multiple sources of exposure from dogs and other animals, human cases are common, despite the utility of India discontinuing nerve-tissue-based products for modern biologics in 2005. Unfortunately, modern biologics used in PEP (i.e., rabies vaccine, RIG, rabies MAbs) are not readily available and are often in short supply [365,366]. Moreover, many medical professionals in these settings have less than ideal knowledge of proper PEP [367]. Dog bite victims or their caretakers, often from remote rural areas, typically must go on a veritable ‘expedition’ to find PEP, resulting in delays or even non-administration of PEP. To help address the issue of poor PEP compliance, an interactive web application, ZeroRabiesApp (ZRA), provides a point-of-care tool for treating dog bite cases [368]. The ZRA is freely accessible via smartphones and computers (https://anyadoc.shinyapps.io/ZeroRabiesINDIA/, accessed on 1 May 2025). This tool provides access to the latest guidelines developed by the WHO and the US Advisory Committee on Immunization Practices. It generates a customized PEP schedule based on the user-provided bite date (day of exposure), which can be shared with the user’s healthcare provider. The ZRA also offers access to a rabies biologics database, which can be used to locate the nearest places where PEP products are currently in stock, ensuring more timely access to necessary care.

### 9.3. Africa

Despite the availability of effective preventive measures since the mid-20th century, tens of thousands of estimated human deaths still occur annually, with 95% of rabies cases in Africa, second only to Asia [333,362]. While a global focus has predominated on eliminating dog-mediated rabies through mass vaccination, human PEP, and public awareness campaigns, other lyssaviruses have been largely ignored [354]. Multiple, intertwined complexities of diverse hosts and pathogens prevail, impacting disease surveillance and control, including the ambitious ZBT goal. While focused on RABV, a need exists for a more inclusive approach for all lyssaviruses, throughout the continent where so-called ‘rabies-related viruses’ (Table 1) were first recognized during the 1950s [29].

Effective animal rabies control relies on robust surveillance systems and targeted interventions [369]. Throughout Africa, few historical examples exist to emulate and are often dependent upon public-private partnerships (e.g., Tanzania) [370]. In areas like KwaZulu-Natal, South Africa, comprehensive strategies—including training, awareness, MDV, vaccine banks, and accessible PEP—led to a temporary decline in human rabies [371]. However, progress was short-lived once international support vanished [372]. In many African countries, surveillance systems remain undeveloped, resulting in underreporting and inadequate control [373]. Strengthening these systems is crucial for accurate data collection and the development of effective response strategies.

Launched in 2015, the ZBT initiative aimed to eliminate dog-mediated human rabies deaths by 2030 [374]. The ZBT strategy emphasizes MDV, public awareness, and accessible human PEP. The basic concept is sound, but the timetable is overly ambitious. In the face of limited continental progress, challenges persist, particularly in regions with limited resources and infrastructure. Achieving the ZBT goal requires prioritization, sustained commitment, reliable funding, and international collaboration. The intense focus on eliminating canine rabies has led to disparities in research and resource allocation. All other lyssaviruses are less studied, resulting in limited understanding and preparedness. This imbalance is evident throughout Africa, where canine rabies is endemic, while other lyssaviruses are scarcely researched. Addressing this gap requires a more inclusive approach that balances efforts between controlling canine rabies as a priority but not ignoring other lyssaviruses within the realm of One Health and concerns about EIDs. Africa bears a significant burden of rabies cases, with estimates suggesting that the continent accounts for approximately 35% to 42% of global-related fatalities [333,362]. What proportion of such suspected human deaths are due to other lyssaviruses besides RABV is unknown, because most cases are not confirmed (or characterized) by laboratory testing. The same is true relative to domestic animal and wildlife cases.

#### 9.3.1. Fragmented Control Programs

Numerous fragmented rabies control programs occur across multiple African countries. Unlike the situation in Europe or the Americas, most are not coordinated through a centralized national rabies control program (NRCP) [375]. This lack of integration results in significant inefficiencies. There is no unified system for monitoring and evaluating prevention and control efforts. As a result, vaccination and education campaigns may be duplicated in communities that have already been covered, leading to wasted resources and efforts. The absence of a designated NRCP further complicates the situation, preventing effective tracking of progress and hindering success by duplicative health initiatives [375]. Establishing an NRCP addresses these challenges in part by integrating the activities of various stakeholders, including NGOs, under a unified strategy. This integration facilitates comprehensive planning, resource allocation, and development of monitoring and evaluation frameworks to assess the impact of control measures.

Besides governmental members, NGOs play a vital role in control by providing resources, expertise, and community engagement. However, without coordination, their efforts overlap, leading to programmatic inefficiencies. For rabies control programs to be effective, countries must take ownership and lead these initiatives. At a minimum, such leadership involves developing national strategies, securing funding, and fostering collaboration among all stakeholders. There is also a high level of dependence on international aid, which is unsustainable in the long term [375]. For example, the recent reductions in international aid by the US administration have highlighted the vulnerability of relying on external support alone for essential health programs. Whereas the African CDC plays a crucial role in broadly strengthening disease surveillance and providing technical assistance, greater investment and a more cohesive strategy are needed at both national and regional levels. Regional cooperation is crucial, as neighboring countries must collaborate to prevent the cross-border transmission of rabies and other diseases. Several Asian countries have successfully implemented integrated surveillance and reporting systems to strengthen rabies control efforts. For instance, Hunan Province in China and Vietnam established comprehensive systems that integrate data collection across human and animal health sectors, facilitating better monitoring and response [283,376]. In addition, India has begun to use standardized reporting formats to help ensure more accurate surveillance and timely intervention (https://rabiesfreeindia.mohfw.gov.in/, accessed on 1 May 2025). However, such integrated systems are largely lacking in Africa, where surveillance remains fragmented and inconsistent, hindering the ability to track and control rabies effectively.

#### 9.3.2. Challenges to Laboratory-Based Surveillance

Weak surveillance systems hinder significantly the detection and understanding of rabies in Africa. For example, one study involving 49 of Africa’s 54 countries found that surveillance systems were ineffective in at least 16 of 23 (70%) respondents to the survey [377]. While most cases are thought to be due to RABV, inadequate diagnostic capabilities contribute to the overall underreporting of all lyssavirus infections. This deficiency leads to imprecise estimates of the human burden and obscures the true epidemiology of the disease [302,378]. Financial constraints further exacerbate surveillance limitations. In Madagascar, inadequate funding hampers access to suspected animals, proper restraint, sedation and euthanasia, collection of brain samples, and the implementation of necessary biosecurity measures, resulting in significant underreporting [379]. Such shortcomings impede effective monitoring, underscoring the need for strengthened surveillance systems, enhanced diagnostic capacities, and adequate support to assess the actual status of rabies. Even in the face of such limitations, practical disease control and introspective pathogen discovery are not mutually exclusive, as seen with concomitant rabies prevention and epidemiological surveillance, focused upon several lyssaviruses (e.g., DUVV, LBV, MOK, RABV, etc.) within South Africa, including identification of RABV reservoirs among multiple wild mesocarnivores, including aardwolves, foxes, jackals, mongooses, etc. [65,92,100,107,108,148,169,380].

#### 9.3.3. Consumption of Infected Animals?

Rabid animals should not knowingly enter the food chain. While livestock are generally dead-end hosts, sick animals are slaughtered and enter local markets [381,382,383]. Such events create a risk for viral transmission through salivary contamination of open wounds and exposure to infected tissues during the preparation of animals for human consumption. These practices complicate surveillance and response, as butchers often avoid testing animals due to their economic value, further hindering accurate disease forecasting. While exposure to infected animal products is not a common route for productive infection, and proper cooking/pasteurization inactivates lyssaviruses present within tissues, there are rare cases where ingestion could pose a risk if the animal was in an advanced stage of rabies, and the ensuing creation of public anxiety in a need for health assessments and the procurement of limited PEP [272]. Additionally, besides meat, there are many reports of individuals consuming raw cow milk. In Zimbabwe, cattle owners may be unaware of zoonoses [384]. If milk is consumed from a cow bitten by a rabid dog, and the cow dies from rabies, village members may be placed under surveillance due to concerns over potential viral exposures. Information alone may not outweigh risky traditional practices. As evident in a survey from Ethiopia, >75% of respondents had a good perception of zoonotic disease transmission, but the practice of consuming raw milk or raw/undercooked meat was still high [385]. Similarly, survey participants in Guinea may have understood the dangers they undertook daily, based on the values placed upon livelihoods dependent upon animals, but expressed a sense of powerlessness over an uncertain world, emphasizing that ‘…zoonotic disease prevention demands a thorough and culturally nuanced understanding of the factors that influence preventive behaviors’ [386]. Beyond domestic animals, consumption of bushmeat is a common practice in Africa. In one survey from Malawai, at least 50% of participants consumed bat meat [387], illustrating potential zoonotic risks on a regular basis among certain rural communities.

#### 9.3.4. Veterinarians at Risk of Viral Exposure

Local veterinarians who vaccinate and act as caregivers for sick animals are at risk of viral exposure. Unfortunately, there are limited data on the percentage of African veterinarians who are recipients of PrEP. One study at the University of Ibadan in Nigeria found that 15.4% of veterinary students had the recommended PrEP regimen. Approximately 19% received only a single dose. This indicates that while awareness of rabies may be relatively high among some professionals, the actual uptake of PrEP remains low, likely due to barriers such as access, cost, and awareness [388]. To expect that veterinary professionals will remain engaged in relevant disease surveillance and control practices may be unrealistic if vaccination is not more widely available and provided for free within enzootic LMICs. New simplified dose-sparing methods, providing for vaccination in one week, rather than 3 weeks or more, may offer greater opportunities for key professionals across the globe [389,390,391].

### 9.4. North, Central, and South America

The Americas is the only region where the sole lyssavirus representative is RABV, with more indigenous viral variants present than the number of recognized lyssavirus species [392]. The MDV programs began during the 1920s and gradually North America eliminated canine RABV transmission [242,393,394]. Similar progress is occurring increasingly in Central and South America [353,395]. Currently, foci of canine RABV circulate in Bolivia, Haiti, Venezuela, and most recently in Peru [396,397,398,399,400]. Besides dogs, major RABV reservoirs reside among wildlife, including wild mesocarnivores, non-human primates (e.g., marmosets in Brazil), and multiple species of frugivorous, hematophagous, and insectivorous bats [401]. Uniquely, viral spillover infections and host shifts are evident from bat RABV to mesocarnivores [242,402,403,404]. Both Canada and the US utilize oral vaccination to control rabies in coyotes, foxes, or raccoons [271]. On the Caribbean islands, rabies occurs in Cuba and Grenada (e.g., in mongoose predominantly, and bats), Hispaniola (e.g., via dogs and mongoose), Puerto Rico (e.g., via mongoose), and Trinidad (via vampire bats, *Desmodus rotundus*) [405] In addition to enhanced surveillance, antigenic typing and molecular characterization provides an appreciation of the diversity of RABV variants among different hosts, as found with diverse mesocarnivores (irrespective of many more in bats) within a single country, such as the US [242].

### 9.5. The Caribbean, with Special Reference to Trinidad: Cattle, Small Ruminants, and Water Buffalo

Globally, livestock deaths due to rabies have been estimated at >$500 million USD per year, with major losses in countries with livestock-dependent economies [333]. While the impact is substantial to these countries, this loss represents only 6% of the global economic burden of rabies. Geographical differences in animal reservoirs necessitate more tailor-made strategies to prevent spillover into livestock populations. Livestock are considered dead-end hosts, but act as sentinels for viral activity. In the New World, countries such as Trinidad (the only Caribbean island with extant vampire bats), have been dealing with the issue of rabies and livestock since the 1930s, when vampire bats were recognized as a reservoir [406,407]. At that point, there was no precedent for managing large-scale outbreaks in livestock populations, given that dogs were still the dominant reservoir in Latin America. Canine rabies was prioritized largely as a public health problem (such as in the Caribbean on Hispaniola), with viral spillover mainly to companion animals and humans. Alternatively, RABV from vampire bats was primarily transmitted to livestock. As such, this New World phenomenon was viewed as an animal production issue, with potential threats to local food security, particularly for the small farmer. Fortunately, due to the recent shift in the disease paradigm in Latin America, there has been a greater impetus for focusing on rabies in herbivores [408,409]. The risk groups for rabies in herbivores were narrower than those for canine-transmitted RABV. These included mainly farmers and butchers, considering that veterinarians and other animal health workers should have PrEP. Therefore, there was a lower risk of spillover infection to humans overall from livestock, in contrast to mesocarnivores and bats. In fact, during the last 5 years, only one reported human case was attributed to viral transmission from a domestic herbivore in the Americas [408]. This case occurred in 2023, from a bovine in Minas Gerais, Brazil [410]. Nevertheless, with reservoir population expansion (such as from climate change), more herbivore-reservoir interactions (e.g., increased vampire bat predation) can potentially multiply this risk, with more rabid herbivores for opportunistic spillovers. With vampire bats as vectors, livestock can act as buffers to human attacks. However, if the livestock population dwindles, humans may become alternate hosts with direct relevance to public health.

In Trinidad, the profile of the livestock industry transformed over time due to multiple factors (e.g., agricultural incentives, market demand, changing land usage, etc.). With this change came an evolution of the rabies risk profile for animals. For example, from 1971–2015, there was a notable increase in small ruminant cases, particularly goats [411]. Consequently, rabies control measures changed. Vaccination of goats is conducted in Trinidad, alongside sheep, as they are now considered higher-risk species for rabies acquisition, due to increased vampire bat attacks. Reporting biases may be demonstrated for these smaller-sized herbivores, as they can either be more easily retrievable for testing (positive bias) or more easily discarded (negative bias).

Water buffaloes (*Bubalus bubalis*), locally referred to as ‘bison’ (distinct from native North American bison) or ‘hog cattle’ were imported to Trinidad from India in two major events during the periods 1905–1908 and 1923–1949 [412,413,414]. They were brought to assist with work in the sugar and coconut industry by hauling carts and plowing fields. Imported breeds included both the swamp- and river-type animals, whose genetic stock was used selectively to produce the ‘Buffalypso’, a unique Trinidadian hybrid specialized for meat production and draught [413,414,415]. These animals were seen commonly throughout the island until the downturn of the sugar cane industry in 2003. Thereafter, water buffaloes declined 58% over two decades [416,417]. This population saw a further 25% reduction by 2012 with 1153 animals country-wide, the majority at three government-owned farms, the largest of which was threatened by a high prevalence of brucellosis [417,418]. The RB51 brucellosis vaccine, efficacious in cattle against *B. abortus* infection and abortion, failed to provide similar effectiveness in Trinidadian water buffalo, despite seroconversion [419,420,421,422]. Lack of a cell-mediated immune response after RB51 vaccination might explain the lack of efficacy [423]. Rabies vaccines are highly efficacious in multiple livestock, although they are not licensed or validated for use in hybrid species. However, given the notable challenges in efficacy for the RB51 vaccine in water buffalo, investigations into the comparative immune coverage provided by rabies vaccination in this species are warranted.

**Figure 11:**
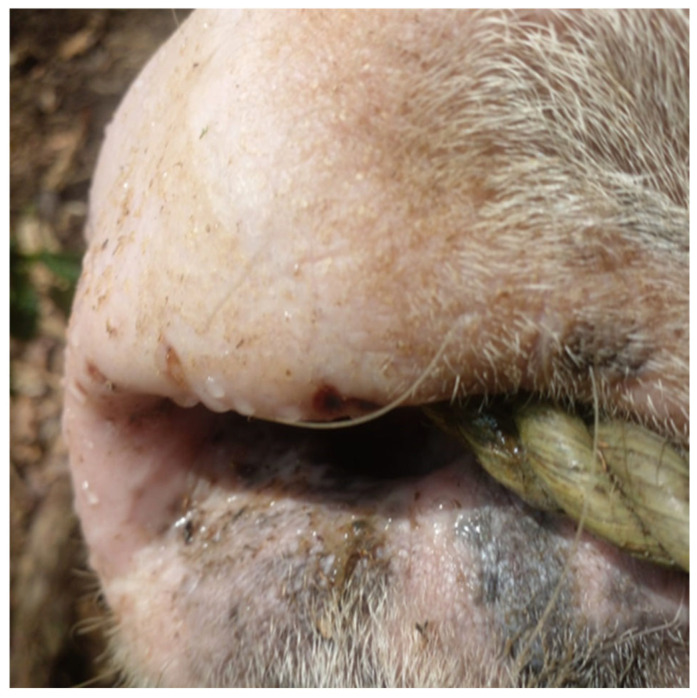
Vampire bats can bite and create lesions on the nostrils of water buffalo.

Among larger herbivores, rabies is less frequently reported in buffaloes than in cattle [424]. Fewer reports are documented from Trinidad, Mexico, Brazil, Ecuador, and Peru in the Americas, as well as from Egypt, India, and China [408,411,424,425,426,427,428,429]. The overall limited detection of rabies cases in buffalos may be due to a combination of factors. In the case of RABV transmitted by carnivores (e.g., dogs), these sturdy animals are believed to be more capable of defending themselves against ground attacks [144]. Although they are less able to physically fend off aerial attacks by bats, they have some anatomical advantages that may lessen bites from these much smaller mammals. For example, there is a notable difference in the skin thickness between buffalo and cattle. Buffaloes exhibit significantly thicker skin in the regions commonly targeted by vampire bats for feeding (i.e., the neck, ribs, back, hock, and tail base). These areas thicken with age [430]. Such features may preclude easy feeding (i.e., biting) by vampire bats. Nevertheless, in a skillful display of ingenuity, vampire bats have been noted to feed on buffaloes by biting their softer nostril skin (Figure 11) when they are on the ground wallowing in mud or in water [431].

**Figure 12:**
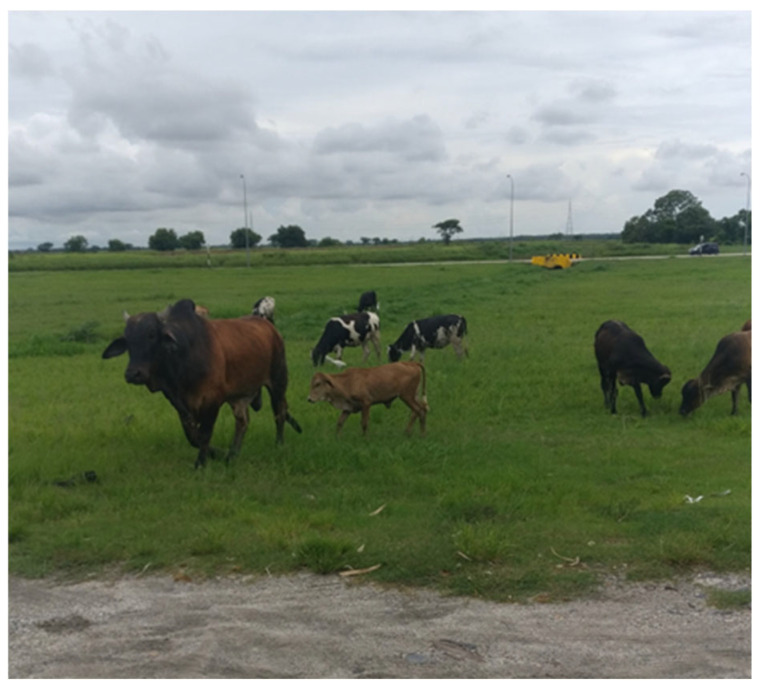
Free-ranging livestock along roadways in Trinidad.

Other factors which may contribute to a lower reporting of cases in buffalo include surveillance bias and the relative inaccessibility of animals for observation and testing, especially with animals on pasture. In Trinidad, water buffalo typically raised under such extensive management conditions are on only a few large farms. Individual farmer holdings generally consist of only 2–5 animals per farm [432]. Many farmers practice pastoral or ‘land-less’ farming, where herbivores are moved from location to location for grazing. In Trinidad, animals are sometimes left to roam freely along roadways during feeding, posing vehicular safety concerns (Figure 12). Another situation on the island is the illegal importation of livestock from the South American mainland. Reports decry large numbers of ‘stray’ animals wandering around villages and towns in the southern part of the country [433,434]. Owners of such animals come forward rarely to report ill or dying animals or request rabies vaccination, due to fear of litigation or persecution.

While extensive or pastoral animal rearing in theory increases wildlife reservoir-livestock interactions and enhances opportunities for virus transfer, concomitantly it decreases opportunities for case detection by limiting opportunities for direct observation of animals. Therefore, due to surveillance and reporting limitations, the true prevalence of rabies in livestock may be grossly underestimated in areas where such practices prevail. Other challenges to management in extensively reared herds include difficulties with animal identification, capture and restraint for rabies vaccination, and clinical engagement. Misdiagnosis of cases may also be possible with limited testing and reportsmay be based on clinical observations alone. Veterinary involvement may not be particularly frequent. Incubation periods in buffaloes may be more variable (2–12 weeks) compared to cattle [144,435,436]. Clinical signs are largely similar (e.g., pica, bruxism, a fixed stare, copious salivation, hoarse bellowing, hind limb paresis, incoordination, paralysis, recumbency, and death within five days of disease onset). However, tail paralysis or a kinked tail that is notable in buffalo has not been documented routinely in other bovids [144,435]. No rabies vaccine is licensed for water buffaloes, although these biologics are believed to be highly efficacious across species. Regardless, administration poses logistical challenges pertaining to the restraint of these highly aggressive animals and vaccination techniques (e.g., needle placement and length) for successful delivery, due to their unique physique.

### 9.6. Australasian Region

Historically, Oceania (consisting mostly of water, with several thousand volcanic islands or atolls), was deemed generally to be ‘rabies-free’ (yet without the enactment of routine laboratory-based surveillance) [437]. However, even there, the epidemiological situation is not static, as was observed in Guam, with disease introduction and control during 1967–1968 [438]. Also, besides dogs, rabid wildlife, such as bats, may be translocated to far-distant islands [439,440].

Seemingly, such Pacific outbreak rarities, relative ‘splendid isolation’ between sources and sinks, the long temporal expanse from the first and only suspect ‘outbreak’ during 1866–1867, and the enactment of a strict quarantine, lulled Australia into the belief that rabies was absent [441,442]. In a comparison of that continent to the enzootic situation in the US at that time, Skerman (1964) mused that apparently ‘… conditions for the establishment of permanent reservoirs of infection do not exist…’ [443]. On the legitimate risk of rabid dog importation from the Southeast Asian region, some opined that ‘…it would be foolish to say that it cannot happen here…’, balanced with ‘…the most we can say is that it need not happen here.’ [444]. Concern for introduction was warranted, because if dog rabies entered and became enzootic, investigators estimated that >35,000 bites might require PEP [445]. From a travel medicine lens, professionals warned that ‘…sooner or later human rabies will occur…through the arrival in Australia of a person who has been infected with the virus in another country.’ [446]. On the question of such ‘remote unlikelihoods’ (like organ transplant cases), attention dawned on some physicians regarding the topic, that because ‘…encephalopathies resembling rabies are seen rarely enough, all possible causes of obscure neurological illnesses should be considered.’ [447].

In a somewhat prescient fashion, in 1987, the case of a 10-year-old Australian boy was diagnosed with rabies—four months after death [448,449]. He had traveled throughout Asia with his mother (and may have been infected after a monkey bite in India, 16 months before onset), becoming ill some 8 months upon his return. Clearly, this unique case demonstrated ‘…the need to include rabies in the differential diagnosis of encephalitis…’, with a concurrent understanding that ‘…to establish a diagnosis of rabies may be difficult.’ [450]. Thereafter, a second case, diagnosed 3 months after death, occurred in a 10-year-old Vietnamese girl—more than six years after her presumed exposure abroad [451,452] As stated by Grattan-Smith, ‘…rabies occurs in Australia and needs to be considered in the differential diagnosis of acute encephalitis…’ [453]. Slowly, even after these unusual human cases, only a minority believed ‘it could happen here’, although apparently still focused on the obvious major threat posed outwardly by canine rabies, but not really from within [454,455].

**Figure 13:**
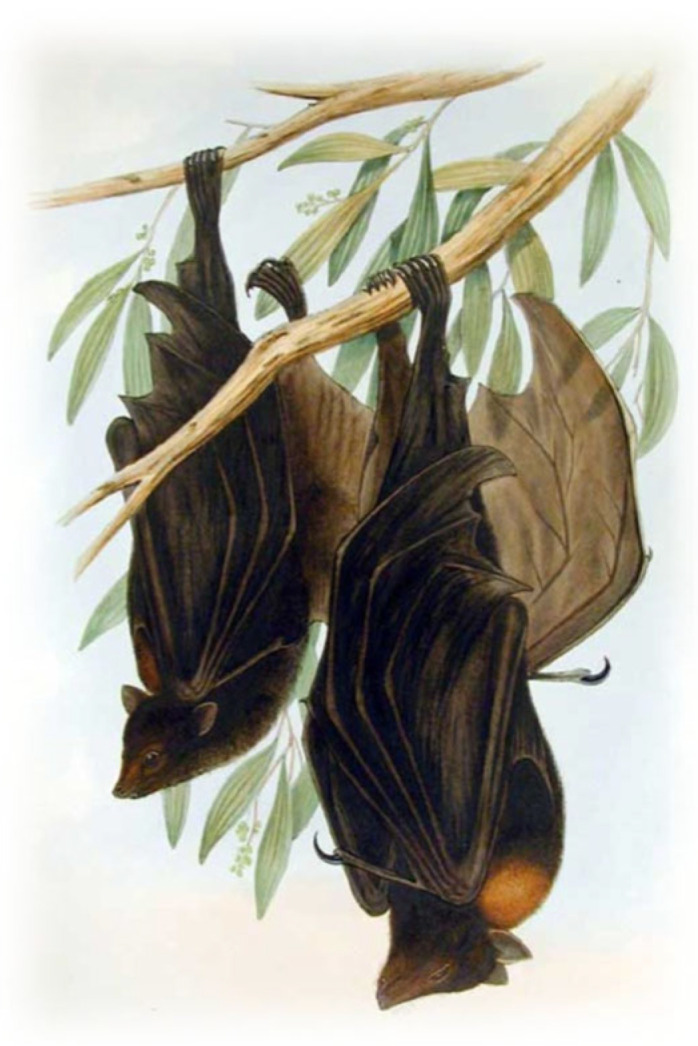
Artistic depiction of a black flying fox, *Pteropus alecto*. (Courtesy J. Gould, Mammals of Australia, 1863, Wiki Commons, https://commons.wikimedia.org/wiki/File:Pterogus_alecto1.jpg, accessed on 19 May 2025).

The discovery of enzootic rabies happened only via an unrelated EID event. In 1995, after an equine morbillivirus (AKA Hendra virus today) outbreak among horses (with associated human cases), Australian investigators began a search for apparent reservoirs involved in viral emergence among suspected wildlife [456]. As part of that epidemiological inquiry, in 1996, a juvenile black flying fox (*Pteropus alecto*) in Ballina, New South Wales was euthanized, after being found under a fig tree, unable to fly. This species is the largest bat in Australia, roosting in noticeable ‘camps’ consisting of hundreds to thousands of individuals (Figure 13). The necropsy revealed a severe nonsuppurative encephalitis [54]. Histologically, eosinophilic, intra-cytoplasmic inclusions were present within neurons. Electron microscopic images of the CNS displayed aggregates of viral nucleocapsids within the cytoplasm of cell bodies. Within the brain, an immunohistochemical test for rabies was positive, as was a similar finding in a retrospective bat CNS from 1995, with multiple representative intracytoplasmic inclusions (Supplementary Figure S4).

After the passage of infected tissues in laboratory mice, examination of brain homogenates by electron microscopy revealed bullet-shaped particles, typical of rhabdovirus virions. Phylogenetic analysis showed that the virus associated with the bats was closely aligned with classical RABV. Antigenic typing with a panel of MAbs confirmed that the bat viral isolate was a lyssavirus, different from others in the genus, but very similar to RABV.

The bat viral isolate was neutralized by anti-sera to RABV. Provisionally, the virus was called ‘pteropid lyssavirus’, before being termed ABLV. Besides diagnosis in *P. alecto*, ABLV was also identified in the little red flying fox (*Pteropus scapulatus*) from north Queensland [457]. Specific diagnostic tests were necessary for definitive diagnoses of lyssavirus infection rather than dependence upon clinical signs or histology alone, because of multiple factors involved in bat morbidity and mortality, such as toxicity [458]. Further phylogenetic analyses supported the concept that ABLV was a new member of the *Lyssavirus* genus [459,460].

As surveillance in bats increased to better understand the epizootiology of ABLV, prior prophylaxis recommendations for travelers evolved to help safeguard the health of exposed individuals within the country [461,462,463]. Gradually, other reports of ABLV in bats grew to include the Northern Territory, Queensland, New South Wales, and Victoria. Most of the initial bat sampling was opportunistic, and focused on eastern Australia. To detect prior deaths from rabies, one retrospective epidemiological study reviewed cases of unexplained human encephalitis in the Northern Territory from 1992–1996. The aim was to test any available clinical specimens for evidence of lyssavirus infection and to survey the use of diagnostic testing by clinicians [464]. Unfortunately, only two samples remained (both testing negative). That review found a considerable proportion of unexplained illness among cases of encephalitis but was not able to exclude the involvement of ABLV in affected humans. Resulting recommendations suggested that clinicians should test for lyssavirus infection among encephalitic patients, seek postmortems when human deaths were unexplained, and store appropriately any specimens to enable testing for EID.

Within months of the 1996 discovery, the first recognized case of human rabies due to ABLV was reported in a 39-year-old Queensland woman, who died of encephalitis after bat exposure, acquired during her avocation as a bat carer [465,466]. Rather than from a pteropid bat, apparently an insectivorous emballonurid species, the yellow-bellied pouched or sheathtail bat (*Saccolaimus flaviventris*), bit her (Supplementary Figure S5). Approximately a month after exposure, her presentation was akin to classical rabies (somewhat curiously, the neurological report entitled the case as ‘non-rabies lyssavirus human encephalitis from fruit bats: Australian bat Lyssavirus (pteropid Lyssavirus) infection’). The patient died ~20 days after onset. Although the sequence of this human isolate was believed initially to be most similar to that obtained from pteropid bats, it was later identified as a variant associated with *S. flaviventris* [459]. Given growing public health concerns, in 1997 the provision of rabies PEP for persons exposed to bats in Australia, as well as PrEP for persons with ongoing contact with bats, became consistent with those for travelers exposed to RABV abroad [467]. Nevertheless, public demand for prophylaxis in Western Australia rose only marginally, after initial recognition of ABLV.

During 1998, a second death occurred, in a 37-year-old Queensland woman [468]. In August 1996, 27 months prior to her diagnosis, the woman was at an outdoor barbecue. A flying fox landed on the back of a child and the woman was bitten during her removal of the bat. Within 48 hr, she received antibiotics and tetanus toxoid from her physician (but not PEP!?). Six months later, she inquired about a test for the ‘bat virus’. Although counseled to receive PEP, she declined. She died in December, 19 days after illness onset. Her ‘…clinical presentation, duration, and course…were virtually indistinguishable from those seen in rabies’, as were the postmortem histological findings, including Negri bodies [468]. Initial sequences of an amplicon produced from her saliva indicated infection associated with ABLV but differed somewhat from others available at that time from the reference laboratory. Later analyses of CNS isolates obtained after passage in cell culture confirmed infection due to a *Pteropus* variant of ABLV [469]. After her long incubation period and eventual diagnosis, the child and other exposed individuals at the party received PEP. With sensational media headlines hyping ‘killer bats’, public demand for prophylaxis rose dramatically [470].

A retrospective study of the tissues from 37 naturally infected bats found that the primary lesions in the CNS due to ABLV were identical to classical RABV, as predicted from an examination of other lyssavirus infections [471]. Ongoing surveillance supported the concept that both frugivorous and insectivorous bats throughout Australia were potential ABLV reservoirs, even though laboratory detection of infection in submitted bats remained low, as expected from bat rabies surveillance globally [472,473,474]. Regarding perpetuation, within pteropid colonies especially ‘…large seasonally nomadic, multispecies colonies in which flying foxes commonly congregate (and interact) provide opportunity for interspecies and interregion transmission of ABLV…’ [474].

Acknowledgment of such rabies cases due to ABLV continued to create challenges in both public health and wildlife conservation. For example, the findings of a naturally infected juvenile flying fox placed into captive rehabilitation, with subsequent exposures to unvaccinated persons during continued care, obviated the need for improving community and professional awareness of the disease and associated risks due to such bat exposures [475]. As anticipated, veterinarians were included in the broader at-risk groups for the reception of PrEP as a precautionary measure [476]. It became increasingly apparent that the basic disease caused by infection with ABLV, as well as its primary methods of diagnosis, prevention, and control were not different essentially from RABV and other Phylogroup I lyssaviruses in bats [477,478]. Moreover, it was suspected that although only recognized during the late 20th century, ABLV was likely enzootic in Australia for centuries, predating European colonization [469]. Further studies suggested that ABLV, like RABV, was subject to ‘…strong selective constraints,…a stability of host species, cell tropisms, and ecological conditions.’ [479]. Novel management strategies suggested bat vaccination, but such proposals were never implemented [480,481]. Despite compelling objective data to the contrary, a somewhat confusing public health fact sheet stated ‘…rabies is an infection of mammals that are able to bite and scratch. While it occurs in many parts of the world, it does not occur in Australia…’ [482].

Beyond the deaths of two humans, all cases of ABLV infection from 1995–2005 were detected in bats only. The likelihood of spillover to other animals was considered low. Globally, most rabies cases associated with bat lyssaviruses are intraspecific (Figure 4). Despite the broad host range of RABV, cases of spillover infection from bats to dogs and cats are infrequent, compared to acquisition via rabid mesocarnivores [103,242,483,484]. In Australia, susceptibility testing of small numbers of cats and dogs under laboratory conditions resulted in only limited, non-fatal infection to a single isolate of ABLV [485]. Public health recommendations after Australian dog and cat exposures to bats included close observation for 3 months [486]. Monitoring from 2000–2005 found only 5 instances of dogs with putative contact with rabid bats. All such dogs remained normal after 90 days. However, in 2004, a dog had contact with an untested bat during January, exhibited acute behavioral changes in April, and savagely attacked a child. The dog was destroyed without being tested. With an abundance of caution, the child received PEP. A favorable review of human prophylaxis in the decade after ABLV discovery resulted in a revision of the timing to initiate vaccination. With the reliability of diagnostic testing, delays for up to 48 h were considered acceptable while awaiting results [487]. A review of PEP practices in the Sydney area during 2005–2007 found reasonable patient compliance, but administration did not always occur within the stipulated time [488].

Rabies in humans is rare compared to other neurotropic etiologies (e.g., herpes virus) but should be included in the differential diagnosis of human encephalitis. Nevertheless, the resolution of such cases remains a challenge [489]. One national review of encephalitis-associated deaths in Australia from 1979–2006 found that the proportion of unknown causes rose from 47% during 1979–1992 to 57.2% from 1993–2006 [490]. Presumably, the lack of additional human cases from ABLV from 1998 to 2010 was thought to be related to the implementation of broader PEP measures for exposed individuals, although this lyssavirus was felt to ‘…always have an important part to play in the health of Australians as the density of the human population in Australia and, consequently, the level of interaction between humans and flying foxes increase.’ [491]. Public education and offering of PrEP to professional bat carers and volunteers were important parts of health promotion. A cross-sectional analysis of individuals receiving PEP in New South Wales during 2007–2011 showed that in Australia most exposures were to people who attempted to rescue bats [492].

In 2013, a third human case occurred, in an 8-year-old Queensland boy [493]. In November 2012, while on holiday in the Whitsunday Islands, he was scratched on his forearm by a flying fox. Despite widespread public health communications about the dangers of bat contact and the need for immediate medical assessment in the event of exposure, he did not receive PEP. Apparently, his siblings and friends knew of the incident, but not his parents. Approximately 8 weeks after exposure, he presented with increasingly worsening fever, anorexia, pain, and behavioral changes and he was hospitalized. Positive findings of lyssavirus infection occurred on day 14 of hospital admission, based on serology and hemi-nested RT-PCR. Genetic sequencing revealed a match with pteropid ABLV. His clinical course ‘…followed the pattern of encephalitic rabies…’ and he died in February 2013, 28 days after illness onset [493]. Concerningly, a 2011–2013 survey in New South Wales found that participants ‘…reported a general lack of awareness about ABLV, particularly the risk of disease…’. [494].

Also in 2013, two cases were reported in domestic animals, both unvaccinated horses infected in the same paddock, probably due to exposure to a single infected bat [55]. In that communication, reports of anecdotal cases with similar clinical presentations were mentioned, but ABLV was neither considered nor laboratory testing performed. Since those two unrelated mortality events, no further cases of ABLV have been reported in non-bats to date. Promotion towards greater public awareness, outreach to rural communities, improved access to PrEP and PEP, adherence to best practices in wildlife conservation, continuing professional education among infectious disease specialists and veterinarians, and applied monitoring and research are some of the key interconnected measures to prevent ABLV infections in a One Health context [495,496,497,498,499,500,501,502].

In retrospect, the eventual appreciation by some of rabies in Australia >25 years ago, with different viral variants of ABLV maintained by diverse bat reservoirs, raises intriguing but unanswered questions. Were any human cases unrecognized in the preceding decades? What is the relationship between indigenous First Nations people and wildlife, such as bats (or Bangu to the Yuin)? Are health disparities evident as regards educational outreach and testing? Are native marsupials susceptible? How is the surveillance effort on other taxa, including highly susceptible but introduced mammals, such as red foxes? Was there a rationale for initially restricting domestic animal vaccination? Is there an inapparent viral-species barrier maintained to limit spillover cases in cats and dogs, however rare? Does vaccination status minimize the engagement of more immunologically naïve veterinarians and their staff in patient evaluations with suspect encephalitis and necropsy performance? If the disease was discussed more openly as ‘rabies’, would more veterinarians receive PrEP? What is a so-called ‘rabies-like’ disease that is often cited, considering objective criteria? Has the emphasis on Hendra virus brought any untoward confusion in the general population about rabies, bats, and ABLV and created any challenges to balance negative attitudes with conservation benefits? How far does ABLV extend outside of Australia to other bat populations in Asia or Oceania? Answers to such questions provide a cautionary tale about the existence of lyssaviruses elsewhere, with less refined surveillance systems and public health/veterinary infrastructure, to more fully recognize a virulent (albeit neglected) vaccine-preventable disease, caused by RABV, ABLV, and their Phylogroup I viral relatives.

## 10. Bats, Lyssaviruses, and Serology

Some epidemiologists in so-called ‘rabies-free’ regions may harbor an opinion to the effect ‘…I would recognize rabies, if it was present…’. Professional opinions aside, multiple epidemiological events prove otherwise. When rabies occurs in domestic species, such as dogs, human cases typically follow suit, but are often misconstrued, especially after an underappreciated translocation event (e.g., to islands, such as Bali). If wildlife rabies predominates, without the obvious spillover infections to domestic species commonly, apparent ‘epidemiological silence’ presides, where the disease is functionally an abditive condition, due to obvious surveillance bias. Clearly, the usual suspects do not appear in a differential diagnosis list, when they are not believed to occur indigenously. Such a notorious precedent is exemplified by the eventual detection of RABV in ferret badgers and TWBLV (on one medium-sized island) or ABLV (on an entire continent), both in localities with above-average public health systems.

Beyond mere clinical suspicion, epizootiological surveillance of lyssavirus activity may occur through laboratory confirmation via the detection of antigens, amplicons, or antibodies. For the latter, one requires either neutralization testing (e.g., FAVN, RFFIT, etc.) using one of the known lyssavirus species or in vitro tests, such as ELISA (provided that suitable analytes are available). On every continent where the Chiroptera are found, rabies cases appear in humans and domestic animals (Table 1). As such, bats are one of the most common mammalian groups used in such serological surveillance studies, due to the large number of lyssaviruses associated with the Chiroptera, their immunological reactivity, and the likelihood of abortive viral infections. Serological techniques are useful, because blood samples may be obtained safely without harm to the bats. From longitudinal studies of several years duration, the detection of such antibodies is interpreted as exposure to a given viral variant and not indicative of being rabid [503]. As there are various serogroups with cross-reactivity among shared viral epitopes, if positive bat sera occur when using ‘lyssavirus x’ as an analyte, the conclusion is not necessarily that ‘lyssavirus x’ is present. Rather, it, or a related member, is responsible for the exposure in that host. For example, sera against RABV will cross-react against ABLV, DUVV, etc., and one cannot conclude that RABV perpetuation is operative, as opposed to another phylogroup member. If no lyssaviruses in phylogroup II or III are used, no conclusion is possible about the existence of members in those groups (or other members or groups that await discovery, as one is limited by the unknown). In geographic areas where only one lyssavirus perpetuates, surveillance is more simplified, such as in the New World, because RABV is the only lyssavirus in the entire region based upon decades of surveillance. However, serological surveillance is complicated in part, not only because of the necessity for the proper viral agents of interest, but also the small volume of sera that may be obtained from most bat taxa. In addition, one requires positive and negative control sera and criteria for the cutoff ranges used. Globally, many studies have found positive bat sera indicative of lyssavirus activity (Supplementary Table S5).

Such antibody persistence against lyssaviruses in bats may be life-long [521]. Moreover, antibody dynamics in a single host may vary depending on the lyssavirus variant. For example, in Nigeria, samples collected from *Eidolon helvum* fruit bats, captured for human consumption, showed varied neutralization, based on sublinage: LBV-A (63%); LBV-D (49%); LBV-C (45%); and LBV-B (24%). These data suggest that exposure to LBV is common [522]. The incorporation of information on the distribution of various species is useful for consideration as to which lyssaviruses may circulate in different taxa. For example, the broad Old World distribution of *Pteropus* spp. (AKA flying foxes) and their epidemiological association of ABLV, GBLV, etc., provides a credible rationale for serological surveillance for these and other lyssaviruses as a starting point to consider epidemiological introspection within Indonesia, Madagascar, Malaysia, Oceania, Southeast Asia, etc.

Human exposure may be higher to wildlife hunted as bushmeat, as the larger pteropids are valued for consumption as well as their mystical properties by some cultures, such as in parts of Australia [523]. Even smaller species may be included in tribal festivals, such as in parts of Africa [524]. Other widespread taxa for potential disease ecology considerations might include only those broadly distributed and abundant (i.e., non-endangered, of least concern status for non-lethal methods of serological sampling) species representatives within *Eidolon*, *Epomophorus*, *Eptesicus*, *Miniopterus*, *Myotis*, *Nycteris*, *Rousettus*, etc.

When routine surveillance activities for lyssavirus detection in domestic animals or in wildlife, such as bats, are lacking, risk modeling is another approach for localities that consider themselves ‘rabies-free’, while lacking the laboratory-based criteria for such verification, to consider for prioritization [525]. As serological detection provides evidence of viral activity at orders of magnitude higher than detection of either antigens or amplicons (i.e., <1/100–1/1000 positives per number of samples) within affected populations, from sample sizes of ~30 individuals at a minimum (based upon seroprevalence found globally), such data accumulation via longitudinal studies of highly abundant species provide a reasonable consideration for long term ecological monitoring.

## 11. Current and Future Concerns

Lyssaviruses are widely distributed on all continents, except Antarctica (but even that continent is at risk of potential infection among diverse marine mammals, given a recent major outbreak among African fur seals). These diverse RNA viruses all cause acute progressive encephalitis, typically transmitted via a bite from an infected, rabid mammal (even though birds are also susceptible). Despite historical recognition and relevant exemplification as a One Health priority, this singular zoonosis continues to impose a substantial burden on agriculture, public health, and conservation biology. In addition, virological confusion appears when the historical difference between ‘etiology’ and ‘disease’ goes unappreciated. Understandably, academic consternation abounds if investigators mistake epidemiological terminology focused on local control, regional prevention, or selective elimination with true ‘eradication’. Similarly, challenges can arise if certain political units believe they are ‘disease-free’, yet some international criteria for objective laboratory-based surveillance are unmet, especially in the face of the cryptic existence of likely wildlife reservoirs. With a basic understanding of related pathobiology since the 19th century, the presence of improved sensitive and specific diagnostic tests ever since Negri/Williams, and the availability of increasingly safe and effective biologics from the 1920s to date, it seems rather curious that these pathogens and this disease remain neglected, even with the mindful impact of 20th-century tools. This is not simply a focus on the situation within LMICs, as a brief reflection on recent US surveillance data illustrates this concern.

Before the control and elimination of canine rabies, most human cases were due to rabid dogs. Today, human cases in the US are rare, usually associated with bat RABV infections or due to imported cases exposed abroad [526]. In 2019 and 2020, no human cases of rabies were reported, in contrast to the five deaths in 2021 [527]. Thereafter, no human rabies deaths were reported in 2022 or 2023 [242]. Yet, in 2024, 5 cases were reported: a suspected human rabies death in the Panhandle region of NE; a CA teacher bitten by a bat in her classroom; an elderly woman in MN exposed to a bat; a person in KY believed to be exposed in the Caribbean; and an individual who died during December, serving as an organ donor for a transplant recipient in Michigan, who subsequently succumbed in 2025 [528,529,530,531,532]. Such a veritable epidemiological footnote reveals the biology of rare events in a highly developed, dog rabies-free country and the unpredictability of when, where, how, or why a lyssavirus infection will present.

## 12. Conclusions

We have demonstrated that the zoonosis rabies, as illustrated by some Phylogroup I lyssaviruses, is amenable to control, prevention, or selective elimination (i.e., of a few variants of certain viral species), but this disease is not subject to eradication. Rather, with an abundance of caution related to the same malady caused by multiple related pathogens, we recommend strongly that all veterinary professionals: differentiate etiology from disease; receive PrEP; administer approved vaccines to any domestic animals at risk of exposure; understand the public health concerns posed by lyssaviruses (and that the disease is incurable); recognize the clinical signs in suspect patients; restrain, sedate, euthanize and collect appropriate brain tissue for testing; adhere to biosafety practices; use PPE; report positive case findings for requisite notification; counsel any potentially exposed clients on the need to seek prompt medical attention, including consideration for PEP; appreciate that some animal reservoirs are as of yet unknown; perceive that localities may not actually be ‘free’ of a ‘disease of nature’ without considerable objective diagnostic introspection; and encourage national authorities to incorporate pathogen discovery and viral characterization as a routine part of modern laboratory-based surveillance.

Additionally, from the basic perspective of veterinary virology, all is not resolved. In 2025, there is no known limitation to mammalian host breadth, no in-depth underlying understanding of the recent virological ‘success’ of RABV in light of its extant relatives, no panlyssavirus biologics, no licensed antiviral drugs, no universal therapy on the horizon, and no academic consensus if substantive host shifts will appear shortly or when ‘new’ lyssaviruses will be appreciated. Under such obvious constraints, current global roadmaps for canine rabies elimination and approaching ‘ZBT’ timetables for substantial improvements seem almost simple by comparison, posing a true transdisciplinary challenge.

## Figures and Tables

**Table 1:** Currently recognized and putative lyssaviruses.

Lyssavirus (Phylogroup)	Common Name	Reservoirs	Year and Localities	Spillover Infections	References
*Lyssavirus aravan* (I)	Aravan virus (ARAV)	Insectivorous bats (e.g., *Myotis blythi*)	1991, Central Asia (e.g., southern Kyrgyzstan)	?	[49,50,51,52,53]
*Lyssavirus australis* (I)	Australian bat lyssavirus (ABLV)	Frugivorous and insectivorous bats (e.g., *Pteropus, Saccolaimus*, etc.)	1995, Australia	Humans, horses	[54,55,56]
*Lyssavirus bokeloh* (I)	Bokeloh bat lyssavirus (BBLV)	Insectivorous bats (e.g., *Myotis, Pipistrellus* spp.)	2009, Europe (e.g., Germany, France, Poland, etc.)	?	[57,58,59]
*Lyssavirus caucasicus* (III)	West Caucasian bat virus (WCBV)	Insectivorous bats (e.g., *Miniopterus schreibersii*)	2002, Europe (e.g., Krasnodar region of Russia, Italy, etc.)	Cats	[60,61]
*Lyssavirus duvenhage* (I)	Duvenhage virus (DUVV)	African bats (e.g., *Nycteris thebaica*)	1970, Sub-Saharan Africa (e.g., South Africa, Kenya, Zimbabwe, etc.)	Humans	[36,37,62,63,64,65]
*Lyssavirus formosa* (I)	Taiwan bat lyssavirus (TWBLV)	Insectivorous bats (e.g., *Pipistrellus* spp.)	2016, Taiwan	?	[66]
*Lyssavirus gannoruwa* (I)	Gannoruwa bat lyssavirus (GBLV)	Fruit bats (e.g., *Pteropus* spp.)	2014, Sri Lanka	?	[67]
*Lyssavirus hamburg* (I)	European bat lyssavirus 1 (EBLV1)	Insectivorous bats (e.g., *Eptesicus, Myotis, Rhinolophus* spp., etc.)	1950s, Europe (e.g., Belgium, Denmark, France, Germany, Netherlands, Poland, Russia, Spain, Ukraine, etc.)	Humans, Cats, Sheep, Marten	[68,69,70,71,72,73,74,75,76,77,78,79]
*Lyssavirus helsinki* (I)	European bat lyssavirus 2 (EBLV2)	Insectivorous bats (e.g., *Myotis* spp., etc.)	1985, Europe (e.g., Finland, Netherlands, Switzerland, UK, etc.)	Humans	[43,80,81,82,83,84,85]
*Lyssavirus ikoma* (III)	Ikoma lyssavirus (IKOV)	Unknown	2009, Tanzania	Civet	[86]
*Lyssavirus irkut* (I)	Irkut virus (IRKV)	Insectivorous bats (e.g., *Murina leucogaster*)	2002, Eurasia (e.g., Irkutsk, east Siberia region of Russia, China)	Humans, Dog	[52,60,87,88]
*Lyssavirus khujand* (I)	Khujand virus (KHUV)	Insectivorous bats (e.g., *Myotis* spp.)	2001, Central Asia (e.g., northern Tajikistan)	?	[52]
*Lyssavirus kotalahti* (I)	Kotalahti bat lyssavirus (KBLV)	Insectivorous bats (e.g., *Myotis* spp.)	2017, Finland	?	[89,90]
*Lyssavirus lagos* (II)	Lagos bat virus (LBV)	African bats (e.g., *Eidolon helvum, Epomophorus wahlbergi, Micropteropus pussilus, Rousettus aegyptiacus*, etc.)	1956, Sub-Saharan Africa (e.g., Central African Republic, Ethiopia, Ghana, Guinea, Kenya, Nigeria, Senegal, South Africa, Zimbabwe, etc.)	Cats, dogs, water mongoose	[29,91,92]
*Lyssavirus lleida* (III)	Lleida bat lyssavirus (LLEBV)	Insectivorous bats (e.g., *Miniopterus* spp.	Europe (e.g., Spain, France, etc.)	?	[93,94,95,96]
*Lyssavirus mokola* (II)	Mokola virus (MOK)	Unknown, but small mammals suspected	1968, Sub-Saharan Africa (e.g., Cameroon, Central African Republic, Ethiopia, Nigeria, South Africa, Zimbabwe, etc.)	Cats, dogs, humans, etc.	[30,31,97,98,99,100]
*Lyssavirus rabies* (I)	Rabies virus (RABV)	Bats, mesocarnivores, non-human primates	Believed to be recognized for millennia, throughout human history	In theory, all warm-blooded vertebrates	[101,102,103]
*Lyssavirus shimoni* (II)	Shimoni bat virus (SHIBV)	African bats (e.g., *Macronycteris vittatus*)	2009, Kenya	?	[104,105]
UNCL	Divača bat lyssavirus (DBLV)	Insectivorous bats (e.g., *Myotis capaccinii*)	2014, Slovenia	?	[106]
UNCL	Phala bat lyssavirus (PBLV)	Bat (e.g., *Nycticeinops schlieffeni*)	2021, South Africa	?	[107,108]
UNCL	Taiwan bat lyssavirus 2 (TWBLV2)	Bat (*Nyctalus plancyi velutinus*)	2018, Taiwan	?	[109]
UNCL	Matlo bat lyssavirus (MBLV)	African bats (e.g., *Miniopterus natalensis*)	2015, South Africa	?	[109]

**Table 2:** Rabies status of selected localities globally, as considered by different international organizations.

Locality	WOAH—Rabies Free?	WOAH—Dog Rabies Free?	WHO—Carnivore Rabies Free?	WHO—Lyssavirus Free?
Australia	Yes	Yes	Yes	No
Canada	No	Yes	No	No
China	No	No	No	No
France	Yes	Yes	Yes	No
India	No	No	No	No
Japan	Yes	Yes	Yes	Yes
Nigeria	No	No	No	No
Uruguay	No	Yes	Yes	No
USA	No	Yes	No	No

**Table 3:** General guidance on the use of rabies vaccines in domestic mammals for management against productive lyssavirus infections.

Host Status	Management Considerations
Naïve, non-exposed, healthy	Pre-exposure vaccination (prime, later boost, based upon label recommendations, or 1 year later)
Naïve, exposed, healthy	Euthanize, or strict quarantine for ~3–6 months (may consider postexposure prophylaxis management if vaccines are licensed for such use, or with agricultural and public health approval)
Previously vaccinated, non-exposed, healthy	Booster routinely, annually to triennially, according to vaccine label indications
Previously vaccinated, exposed, healthy	Booster immediately and observe for ~45 days and euthanize if compatible signs of viral encephalitis appear
Pregnant, naïve, healthy	Pre-exposure vaccination ad hoc
Pregnant, previously vaccinated, healthy	Booster during ~3rd trimester
Neonate, healthy, receiving colostrum from vaccinated dam	Pre-exposure vaccination at ~3–6 months
Neonate, healthy, from naïve healthy dam	Pre-exposure vaccination at first health check
Neonate, healthy, from rabid dam	Sedate dam (and later euthanize), consider emergency cesarean section, vaccinate neonate immediately (with a booster at 3 months), etc. using an abundance of caution with adequate PPE
Any animal with compatible clinical signs of rabies, regardless of age or vaccination status	Euthanize immediately and manage potentially exposed litter mates appropriately

## Data Availability

Not applicable.

## Supplementary Materials

The following supporting information can be downloaded at https://www.mdpi.com/article/10.3390/pathogens14060586/s1, Table S1: General examples of different etiological agents and their singular associated diseases [111,112,113,114,115]; Table S2: Selected case examples of domestic, captive exotic, and managed mammals, naturally infected by a diversity of lyssaviruses [55,61,76,88,120,121,122,123,124,125,126,127,128,129,130,131,132,133,134,135,136,137,138,139,140,141,142,143,144,145,146]; Table S3: Diverse epidemiological observations, selected clinical constellations, and potential laboratory findings associated with lyssavirus infections in rabid domestic animals, based upon reported cases [55,131,147,148,149,150,151,152,153,154,155,156,157,158,159,160,161,162,163,164,165,166,167,168,169,170,171,172,173,174]; Table S4. Selected examples of different commercial animal rabies vaccines; Table S5. Selected global examples of serological surveillance for lyssavirus activity within diverse bat populations [56,61,96,498,504,505,506,507,508,509,510,511,512,513,514,515,516,517,518,519,520]; Figure S1: Historically, unusual signs observed in domestic animals, beyond overt aggression or paralysis, included pica, and upon gross necropsy (well before the development of sensitive and specific laboratory tests) the dissected stomach contents of a dog might include a mixture of mucus, fur, wood chips, straw, or other foreign materials, as depicted in the illustration (Courtesy of G. Fleming [184]); Figure S2: Photomicrographic image of a hematoxylin and eosin-stained brain tissue slide, with non-specific histopathologic changes of perivascular cuffing during lyssavirus encephalitis, with accumulations of inflammatory cell infiltrates, including mixed layers of lymphocytes and polymorphonuclear leukocytes (Courtesy of D. Perl, US HHS Public Health Image Library); Figure S3: Photomicrograph of a hematoxylin and eosin-stained brain tissue specimen slide, illustrating a close-up of a single lyssavirus-infected neuron, with the presence of multiple, oval to spherical intracytoplasmic inclusions (2–10 μm in diameter), known as ‘Negri bodies’ (Original image courtesy of D. Perl, US HHS Public Health Image Library, highlighted via arrows by M. Häggström, made available under the Creative Commons CC0 1.0 Universal Public Domain Dedication, https://commons.wikimedia.org/wiki/File:Histopathology_of_Negri_bodies_in_rabies_encephalitis.png, accessed on 19 May 2025); Figure S4: Representative photomicrograph of a slide of ABLV antigens (magenta-stained inclusions) in the brain of an infected, rabid Australian bat (Courtesy P. Hooper, Creative Commons;https://commons.wikimedia.org/wiki/File:CSIRO_ScienceImage_38_Australian_Bat_Lyssavirus.jpg, accessed on 19 May 2025); Figure S5: Mounted specimen of *Saccolaimus flaviventris*, the yellow-bellied sheathtail bat (Courtesy H. Warwick, Museums Victoria, Creative Commons, https://commons.wikimedia.org/wiki/File:Saccolaimus_flaviventris_Museum_Victoria.jpg, accessed on 19 May 2025).

## Abbreviations

The following abbreviations are used in this manuscript:

ABC-ARV	animal birth control-antirabies vaccination program
ABLV	Australian bat lyssavirus
ACIP	Advisory Committee on Immunization Practices
ARAV	Aravan virus
BBLV	Bokeloh bat lyssavirus
CDC	Centers for Disease Control and Prevention
CNS	central nervous system
CSF	cerebro-spinal fluid
DBLV	Divaea bat lyssavirus
DFAT	direct fluorescent antibody test
DRIT	direct rapid immunohistochemical test
DUVV	Duvenhage virus
EBLV1	European bat lyssavirus 1
EBLV2	European bat lyssavirus 2
EID	emerging infectious disease
ELISA	enzyme-linked immunosorbent assay
FAO	Food and Agriculture Organization
G	glycoprotein
GBLV	Gannoruwa bat lyssavirus
IHC	immunohistochemistry
IKOV	Ikoma lyssavirus
IRKV	Irkut virus
KBLV	Kotalahti bat lyssavirus
KHUV	Khujand virus
L	‘large’ or RNA-dependent RNA polymerase protein
LAMP	loop-mediated isothermal amplification
LBV	Lagos bat virus
LLEBV	Lleida bat lyssavirus
LMICs	lower-and-middle-income countries
M	matrix protein
MAbs	monoclonal antibodies
MBLV	Matlo bat lyssavirus
MDV	mass dog vaccination
MLV	modified-live virus
MOK	Mokola virus
N	nucleoprotein
P	phosphoprotein
PAHO	Pan American Health Organization
PBLV	Phala bat lyssavirus
PEP	Postexposure prophylaxis
PrEP	Preexposure prophylaxis
RIG	rabies immune globulin
RNP	ribonucleoprotein
RPA	recombinase polymerase amplification
SHIBV	Shimoni bat virus
TWBLV	Taiwan bat lyssavirus
TWBLV2	Taiwan bat lyssavirus 2
RABV	Rabies virus
VNA	virus neutralizing antibodies
WCBV	West Caucasian bat virus
WHO	World Health Organization
WOAH	World Organization for Animal Health
ZBT	‘zero by thirty’
ZRA	ZeroRabiesApp
