# Supplementary material for: The Challenge of Lyssavirus Infections in Domestic and Other Animals: A Mix of Virological Confusion, Consternation, Chagrin, and Curiosity
Source: Pathogens. 2025 Jun 13;14(6):586. doi: 10.3390/pathogens14060586 (PMC12195927; doi:10.3390/pathogens14060586)
Supplement: Supplementary file 1 [file pathogens-14-00586-s001.zip › pathogens-3656780-supplementary.pdf]

Supplementary Table S1. General examples of different etiological agents and their singular associated diseases.

**Major Group**	**Infectious Etiology**	**Recognized Disease (etymology)**	**Common Definition**	**Reference**
Helminth	*Trichinella spiralis*, *T. britovi*, *T. murrelli*, *T. nativa*, etc.	Trichinellosis or 'trichinosis' (i.e., that is, relating to a process having minute, hair-like worms)	A parasitic disease, caused by infection with nematodes in the Genus *Trichinella*	[111]
Fungus	*Aspergillus fumigatus, A. flavus, A. niger*, etc.	Aspergillosis (i.e., likening to a process having structures resembling an instrument used to scatter 'holy water' in religious services)	A fungal disease, typically affecting the lungs, caused by molds in the Genus *Aspergillus*	[112]
Protozoan	*Cryptosporidium parvum, C. muris, C. hominis*, etc.	Cryptosporidiosis or 'crypto' (i.e., a process characterized with the entry or formation of small, hidden or 'secret' spores)	A parasitic disease, caused by infection with an apicomplexan in the Genus *Cryptosporidium*	[113]
Bacterium	*Mycobacterium bovis*, *M. tuberculosis*, *M. microti*, etc.	Tuberculosis (i.e., an abnormal process or condition, with bumps, lumps, or nodules)	A chronic bacterial disease, most often affecting the lungs, caused by infection with *Mycobacterium* spp.	[114]
Virus	*Alphainfluenzavirus influenzae*, *Betainfluenzavirus influenzae*, *Gammainfluenzavirus influenzae*, etc.	Influenza or 'flu' (i.e., a suggestion 'to flow in', or 'influence', in the sense of an outbreak, as due to external events)	A contagious respiratory disease, caused by infection with specific influenza viruses	[115]

Supplementary Table S2. Selected case examples of domestic, captive exotic, and managed mammals, naturally infected by a diversity of lyssaviruses.

Animal	Location	Comments	Reference
Anteater	USA	Unusual rabies case detected in a captive zoo animal, after wildlife exposure	[120]
Antelope (e.g., kudu)	Namibia	Unprecedented rabies outbreaks associated with infected dogs, jackals, and suggested kudu-to-kudu transmission in areas where animals were managed for hunting purposes	[121]
Bison	North America	Typically, rabies cases occur in these large ungulates due to viral spillover via infected carnivores to animals managed under park or ranch conditions	[122]
Black bear	USA	Rabies-suspect captive animal resulted in multiple human exposures at a 'petting zoo', but later disproven by laboratory testing	[123]
Camels	Jordan	Reports not uncommon in the Middle East, as demonstrated in one convenience sample where 8/156 cases of rabies were detected at slaughter	[124]
Camelids (e.g., alpaca, llama, etc.)	New World	May be infected from rabid dogs, wild	[125]

		carnivores, or vampire bats throughout North, Central, and South America	
Cat	Italy	Felids are representative of lyssavirus spillover infections from reservoirs, such as this reported case of bat-transmitted WCBV, hundreds of km from index case	[61]
Cattle	Russian Federation	Epidemiological foci of rabies representative of broader lyssavirus circulation, such as reports of >6,700 historical cases of rabies over time	[126]
Civet cat	Nigeria	Captive rabies case in a zoo	[127]
Fallow deer	China	More than 20 rabies cases reported in farmed deer	[128]
Roe (and other) deer	England	Historical rabies outbreak of 'hundreds' of animals affected in Richmond Park, with suggestions of deer-to-deer transmission	[129]
White-tailed deer	USA	Cases of rabies in unvaccinated farmed animals infected by wildlife, with possibility of deer-to-deer transmission	[130]
Dog	China (Global)	Although most rabies cases globally are due overwhelmingly to	[88,131]

		RABV infections by variants adapted to dogs (or spillover from other mesocarnivores), different lyssaviruses may be involved (e.g., LBV, MOK, etc.), or as recently reported, a suspected bat-dog-human infection associated with IRKV	
Donkeys	Ethiopia	~19 laboratory-confirmed cases of rabies from 2003-09 in and around Addis Ababa	[132]
Egyptian fruit bats	Denmark	Outbreak of EBLV1 among captive rabid zoo bats	[133]
Elephant	Sri Lanka	Transmission of RABV suspected via infected dogs	[134]
Equids	Australia	Infection of rabid horses reported with ABLV	[55]
Ferrets	USA	>30 rabies cases reported in domestic ferrets from 1958 to date (including MLV vaccine-associated)	[135]
Goats	Sri Lanka	>200 suspected rabies cases reported from 2005-2014	[136]
Guinea pig	USA	Unusual, suspected exposure from a rabid raccoon to a pet guinea pig, while kept outside	[137]
Lion	India	Captive case of rabies reported in a zoo	[138]

Lesser or red pandas	USA	Captive animals at the US National Zoo, infected during mid-Atlantic raccoon RABV outbreak in the mid-Atlantic region	[139]
Pinnipeds	Southern Africa	Unusual RABV outbreak among African fur seals, posing severe challenges for management by marine mammal conservation groups who may be unaware of such infections	[140]
Poultry	India	Fowl bitten by a rabies-suspect free-ranging dog	[116]
Non-human primates	Multiple countries	Several reports of cases in imported captive taxa as well as enzootic foci among rabid marmosets in Brazil	[141]
Rabbit	USA	Unusual case of pet rabbit attacked by rabid raccoon	[142]
Sheep	Denmark	Reports of infections with EBLV1	[76]
Swine	China	Furious signs of rabies and 20/56 pig deaths over 35 days at 1 farm	[143]
Water buffalos	India	Infections suspected from rabid dogs or mongoose	[144]
Yaks	Nepal	Spillover from infected rabid dogs or wild mesocarnivores	[145]

Zebra	Kenya	A young, orphaned, rabid animal at a safari lodge potentially exposed >150 tourists and local staff	[146]

Supplementary Table S3. Diverse epidemiological observations, selected clinical constellations, and potential laboratory findings associated with lyssavirus infections in rabid domestic animals, based upon reported cases.

**Criteria**	**Reference**
Lack of a history of prior vaccination	[147]
Acute death, without obvious premonitory illness	[131]
Pyrexia	[148]
No significant alterations in serum biochemical or hematologic analysis, but abnormal CSF findings possible (e.g., increased protein concentration, mononuclear pleocytosis)	[149]
Use of MRI may demonstrate abnormal hypersignal T2 changes within the hippocampus, hypothalamus, brainstem, and spinal cord	[150]
Head tilt	[151]
Paresis (e.g., focal or generalized)	[152]
Muscle tremors	[153]
Dysfunctional locomotion (e.g., ataxia, ascending paralysis, hindlimb rigidity, incoordination, reluctance to move, lameness, stiffness, abnormal postures, recumbencies, complete decubitus, etc.)	[154]
Congested ocular mucous membranes, engorged jugular veins	[174]

Cranial nerve deficits (e.g., anisocoria, nictitating membrane prolapse, nystagmus, etc.)	[155]
Pharyngeal paralysis (e.g., dysphagia, hypersalivation, activity reminiscent of foreign body obstructions, etc.)	[156]
Trismus	[157]
Hyperesthesia (e.g., excitable, enhanced reaction to sensory stimuli, including light, sound, touch, etc.)	[158]
Varying levels of consciousness, from lethargic to obtunded	[55]
Heightened aggression	[159]
Sudden change in mentation (e.g., irritability, apprehension, restless, intermittent erratic behavior, biting, scratching, teeth grinding or gnashing, tail wagging, disorientation, etc.)	[160]
Unusual sexual arousal	[161]
Altered vocalizations, such as 'bellowing'	[162]
Pica	[163]
Sudden inappetence proceeding rapidly to anorexia	[164]
Cachexia	[165]
Self-mutilation (excessive head rubbing, limb biting, etc.)	[166]
Uncontrolled paddling, seizures	[167]
Colic	[168]

Dehydration	[169]
Progressive deterioration and worsening systemic signs (e.g., vomiting, diarrhea, stranguria, etc.)	[170]
Coma	[171]
Shortened morbidity period of only a few days, prior to death	[172]
At necropsy, typical pathological findings of mild to moderate, diffuse, non-suppurative encephalitis	[173]

Supplementary Table S4. Selected examples of different commercial animal rabies vaccines.

**Product**	**Producer**	**Animal**	**Dose**	**Age**	**Booster**	**Route**	**Adjuvant**	**Vaccine Type**
Advac DERRILER	Adler Animal Health	Cattle	2 ml	3 months	6 months & annually	IM	None	Attenuated Rabies Virus
Zerorab	Afrivet	Dog<br>Cat<br><br>Cattle<br>Sheep<br>Goat	1 ml<br>“<br><br>“<br>“<br>“	3 months<br>“<br><br>“<br>“<br>“	Within next 9 months, then triennially<br>1 year later, then triennially	IM or SC<br>“<br><br>“<br>“<br>“	Aluminum hydroxide	Inactivated
BAYOVAC DERRIENGUE	Bayer de México S.A. de C.V.	Goats<br>Sheep<br>Cattle<br>Horses<br>Pigs	2 ml<br>“<br>“<br>“<br>“	4 months<br>“<br>“<br>“<br>“	Annually<br>“<br>“<br>“<br>“	IM<br>“<br>“<br>“<br>“	None	Live-attenuated rabies virus
Imunovet R	Bio-vet S/A	Dog<br>Cat	1 ml<br>“	3 months<br>“	Anually<br>“	IM or SC<br>“	Aluminum hydroxide	Inactivated
RAI-VET	Bio-vet S/A	Cattle<br>Horses	2 ml<br>“	4 months, & 30 days later	Annually<br>“	IM or SC<br>“	Aluminum hydroxide	Inactivated
BIOCAN R	Bioveta	Sheep<br>Cattle<br>Horse<br>Pig<br>Goat<br>Camel<br>Dog<br>Cat	1 ml<br>“<br>“<br>“<br>“<br>“<br>“<br>“	3 months<br>“<br>“<br>“<br>“<br>“<br>“<br>“	Every 1-2 yr thereafter	IM or SC<br>“<br>“<br>“<br>“<br>“<br>“<br>“	Algedratum	Inactivated
Lysvulpen	Bioveta	Wildlife (e.g., Fox, Raccoon dog, etc.)	Vaccine-laden bait	Free-ranging	Annually or as needed	Oral	None	Live-attenuated rabies virus

IMRAB 3	Boehringer Ingelheim Animal Health	Ferret<br>Cattle<br>Horse<br>Sheep<br>Dog<br>Cat	1 ml<br>2 ml<br>"<br>"<br>1 ml<br>"	3 months<br>"<br>"<br>"<br>"<br>"	Annual<br>"<br>"<br>1 year later<br>&<br>every 3 years thereafter	SC<br>IM or SC<br>"<br>"<br>"<br>"	Yes	Inactivated
IMRAB + POTOMAVAC	Boehringer Ingelheim Animal Health	Horse	1 ml	3 months	Annually	IM	Yes	Inactivated
PUREVAX 3	Boehringer Ingelheim Animal Health	Cat	0.5 ml	3 months	1 year later<br>&<br>every 3 years thereafter	SC	None	Recombinant canarypox virus vector
RABORAL V-RG	Boehringer Ingelheim Animal Health	Wildlife (e.g., Coyote, Fox, Raccoon, Raccoon dog, etc.)	Vaccine-laden bait	Free-ranging	Annually or as needed	Oral	None	Recombinant vaccinia virus vector
ONRAB	CEVA Animal Health	Wildlife (e.g., Fox, Raccoon, Skunk, etc.)	Vaccine-laden bait	Free-ranging	Annually or as needed	Oral	None	Recombinant AdRG1.3 virus
Rabitec	Ceva Santé Animale	Wildlife (e.g., Fox, Raccoon dog, etc.)	Vaccine-laden bait	Free-ranging	Annually or as needed	Oral	None	Live genetically modified attenuated rabies virus vector
RABMUNE	Ceva Saúde Animal Ltda	Dog<br>Cat<br>Cattle<br>Sheep<br>Goat<br>Horse	1 ml<br>"<br>2 ml<br>"<br>"<br>"	4 months<br>"<br>3 months<br>"<br>"<br>"	Annually<br>"<br>30 days and annually thereafter	IM or SC<br>"<br>SC<br>"<br>"<br>IM	Yes	Inactivated
ETADEX	CZ Veterinaria S.A.	Dog<br>Cat<br>Cattle	1 ml	3 months	Annually	IM or SC	Aluminum hydroxide gel	Inactivated
Rabvac 3	Elanco Animal Health	Horses<br>Dog<br>Cat	2 ml<br>1 ml<br>"	3 months<br>"<br>"	Annually<br>1 year later<br>&<br>every 3 yr thereafter	IM<br>IM or SC<br>"	Yes	Inactivated
Rabies Vaccine, Inactivated (Strain dG)	Guangzhou South China Agricultural University Biopharmaceutical Co., Ltd	Dog	1 ml	3 months	Annual	SC	?	Inactivated
RAKSHARAB	Indian Immunologicals Ltd.	Dog<br>Cat	1 ml<br>"	3 months<br>"	1 month later, then annually	IM or SC<br>"	Aluminum hydroxide	Inactivated
ANTIRRABICA BHK	Instituto Rosenbusch S.A.	Cattle<br>Horses	2 ml<br>"	3 months<br>"	Annually<br>"	IM or SC<br>"	Aluminum hydroxide	Inactivated
Cultivac Rabia	Instituto Rosenbusch S.A.	Dog<br>Cat	1 ml<br>"	3 months<br>"	Annually<br>"	IM or SC<br>"	?	Inactivated
Rabdomun	Intervet	Dog<br>Cat<br>Cattle<br>Horse<br>Sheep	1 ml<br>"<br>"<br>"<br>"	3 months<br>"<br>"<br>"<br>"	Annually<br>"<br>"<br>"<br>"	IM or SC<br>"<br>"<br>"<br>"	Aluminum hydroxide	Inactivated

		Goat<br>Pig	“<br>“	“<br>“	“<br>“	“<br>“		
Canine Rabies Vaccine	Jilin Heyuan Bioengineering Co. Ltd	Dog	1 ml	3 months	Annually	IM	Aluminum hydroxide	Inactivated
RABISYVA VP-13	Laboratorios SYVA S.A.	Cattle<br>Dog<br>Cat	1 ml<br>“<br>“	3 months<br>“<br>“	2 doses, 15 days apart, then annually	IM or SC	Aluminum hydroxide	Inactivated
VACINA ANTI-RABICA	Labovet Productos Veterinários Ltda.	Goats<br>Sheep<br>Cattle<br>Horses<br>Mules<br>Dog<br>Cat	2 ml<br>“<br>“<br>“<br>“<br>1 ml<br>“	3 months<br>“<br>“<br>“<br>“<br>“<br>“	30 days, then annually<br>“<br>“<br>“<br>21 days, then annually	IM or SC<br>“<br>“<br>IM<br>IM<br>IM or SC<br>“	Aluminum hydroxide	Inactivated
RAIVA I	Lema-Injex bioLOGIC	Goat<br>Sheep<br>Cattle<br>Horse<br>Dog<br>Cat	2 ml<br>“<br>“<br>“<br>1 ml<br>“	3 months<br>“<br>“<br>“<br>4 months<br>“	30 days, then annually<br>“<br>“<br>Annually<br>“	IM or SC<br>“<br>“<br>“<br>“<br>“	Aluminum hydroxide	Inactivated
NOBIVAC 3-RABIES	Merck Animal Health (Intervet Inc.)	Ferret<br>Cat<br>Dog	1 ml<br>“<br>“	3 months<br>“<br>“	Annually<br>1 year later &<br>every 3 yr thereafter	SC<br>SC<br>IM or SC	Yes	
EQUI-RAB	Merck Animal Health	Horse	1 ml	4 months	1 year and every 14 months thereafter	IM	Havlogen	Inactivated
NOBIVAC RAGE	MSD<br>Animal<br>Health (Intervet)	Cattle<br>Horse<br>Dog<br>Cat	1 ml<br>“<br>“<br>“	6 months<br>“<br>3 months<br>“	Annually<br>“<br>“<br>“	IM or SC<br>“<br>“<br>“	Aluminum phosphate	Inactivated
NOBIVAC T	MSD<br>Tiergesundheit	Sheep<br>Cattle<br>Horse<br>Dog<br>Cat<br>Ferret	1 ml<br>“<br>“<br>“<br>“<br>“	6 months<br>“<br>“<br>3 months<br>“<br>“	Annually<br>Biennially<br>“<br>Triennially<br>“<br>Annually	SC<br>IM<br>IM<br>IM or SC<br>“<br>SC	Aluminum phosphate	Inactivated
ALURABIFFA	Merial	Goat<br>Sheep<br>Cattle	2 ml<br>2 ml<br>2 ml	3 months<br>“<br>“	Annually<br>“<br>“	SC<br>“<br>“	Aluminum hydroxide, saponin	Inactivated
RabEnd	Tangshan Yian Biological Engineering Co., Ltd.							
ERAG333	Pokrov Biopreparation Plant (not currently being produced)	Wildlife (e.g., Fox, Raccoon dog, etc.)	Vaccine-laden bait	Free-ranging	Annually or as needed	Oral	No	Live genetically modified attenuated rabies virus vector
PROVIDEAN ANTIRRABICA	Tecnovax S.A.	Cattle<br>Horse<br>Dog<br>Cat	2 ml<br>“<br>1 ml<br>“	6 months<br>“<br>3 months<br>“	Annually<br>“<br>“<br>“	IM or SC<br>“<br>SC<br>“	Aluminum hydroxide gel	Inactivated
Rai-Vet	Vaxxinova	Cattle<br>Horses	2 ml<br>“	4 months<br>“	30 days later & annually	IM or SC	Aluminum hydroxide	Inactivated

AFTOGAN + RABIES	Vecol	Cattle<br>Buffalo	2 ml<br>“	3-4 months	Every 6 months - annually	IM or SC<br>“	Oil emulsion	Inactivated
Rabigen<br><br>RabatVac Bovis<br><br><br>Rabigen SAG2	Virbac<br><br>“<br><br><br>“	Dog, Cat<br><br>Horse<br>Cattle<br><br>Wildlife (e.g., Fox, Raccoon dog, etc.)	1 ml<br><br>2ml<br>“<br><br>Bait	3 months<br><br>“<br>“<br><br>Free-ranging	Annually<br><br>“<br>“<br><br>“	IM<br><br>“<br>“<br><br>Oral	AlOH gel<br><br>“<br>“<br><br>None	Inactivated<br><br>“<br>“<br><br>Live attenuated rabies virus
DEFENSOR 3	Zoetis	Sheep<br>Cattle<br>Ferret<br>Cat<br>Dog	2 ml<br>“<br>1 ml<br>“<br>“	3 months<br>“<br>“	Annually<br>“<br>Annually<br>1 year later & every 3 years thereafter	IM<br>“<br>SC<br>SC<br>IM or SC	Yes	Inactivated
CORE EQ INNOVATOR + V	Zoetis	Horse	1 ml	4-6 months	Annually	IM	MetaStim	Inactivated

*Data were obtained from a variety of historical sources including academic, regulatory, pharmaceutical, and product publications/websites. Commercial names are used for comparison purposes only, and do not constitute endorsement.

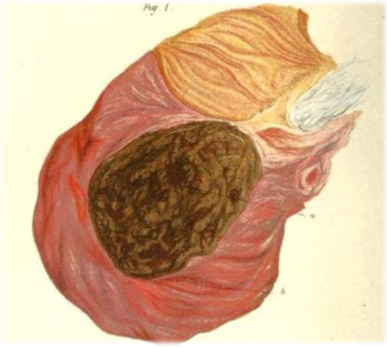

Supplementary Figure S1 . Historically, unusual signs observed in domestic animals, beyond overt aggression or paralysis, included pica, and upon gross necropsy (well before the development of sensitive and specific laboratory tests) the dissected stomach contents of a dog might include a mixture of mucus, fur, wood chips, straw, or other foreign materials, as depicted in the illustration (Courtesy of G. Fleming [184]).

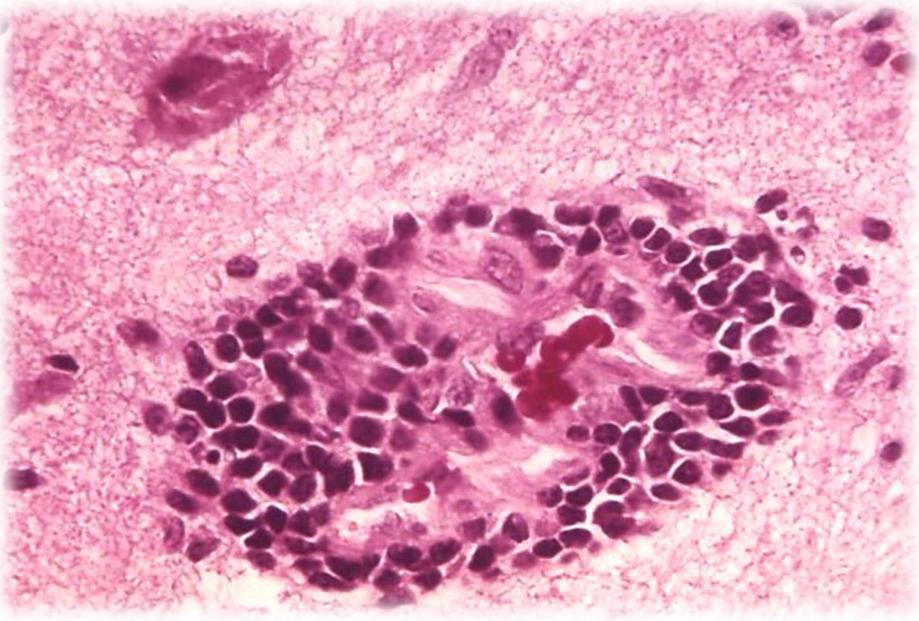

Supplementary Figure S2. Photomicrographic image of a hematoxylin and eosin-stained brain tissue slide, with non-specific histopathologic changes of perivascular cuffing during lyssavirus encephalitis, with accumulations of inflammatory cell infiltrates, including mixed layers of lymphocytes and polymorphonuclear leukocytes (Courtesy of D. Perl, US HHS Public Health Image Library).

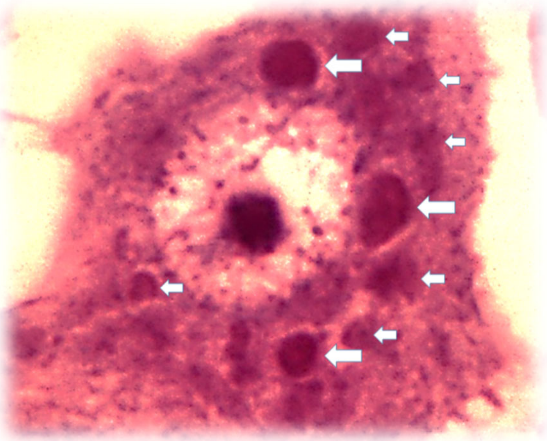

Supplementary Figure S3. Photomicrograph of a hematoxylin and eosin-stained brain tissue specimen slide, illustrating a close-up of a single lyssavirus-infected neuron, with the presence of multiple, oval to spherical intracytoplasmic inclusions (2–10 μm in diameter), known as 'Negri bodies' (Original image courtesy of D. Perl, US HHS Public Health Image Library, highlighted via arrows by M. Häggström, made available under the Creative Commons CC0 1.0 Universal Public Domain Dedication, https://commons.wikimedia.org/wiki/File:Histopathology_of_Negri_bodies_in_rabies_encephalitis.png).

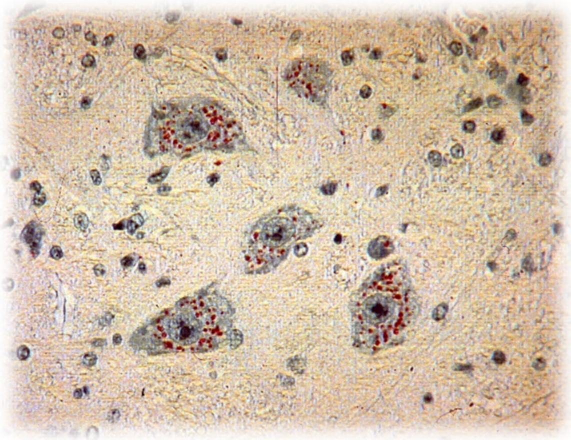

Supplementary Figure S4. Representative photomicrograph of a slide of ABLV antigens (magenta-stained inclusions) in the brain of an infected, rabid Australian bat (Courtesy P. Hooper, Creative Commons;https://commons.wikimedia.org/wiki/File:CSIRO_ScienceImage_38_Australian_Bat_Lyssavirus.jpg).

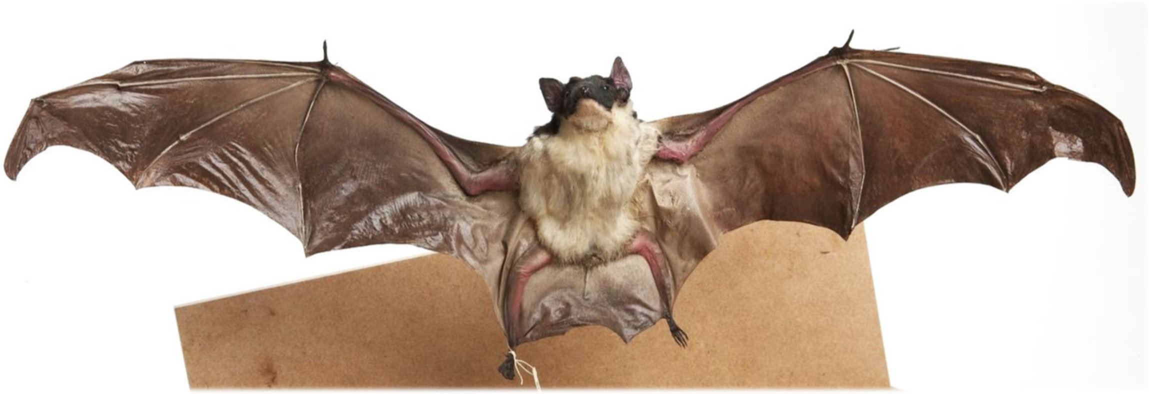

Supplementary Figure S5. Mounted specimen of *Saccolaimus flaviventris*, the yellow-bellied sheathtail bat (Courtesy H. Warwick, Museums Victoria, Creative Commons, https://commons.wikimedia.org/wiki/File:Saccolaimus_flaviventris_Museum_Victoria.jpg)

Supplementary Table S5. Selected global examples of serological surveillance for lyssavirus activity within diverse bat populations.

**Area**	**Bat sampling**	**Comments**	**Reference**
North Africa	693 samples from multiple bat spp.	Of 693 sera, 42 (6%) tested positive for EBLV1–neutralizing antibodies	[504]
Amazonia	307 serum samples, representing 28 bat spp.	156/307 (50.8%) positive samples, the most prominent anti-	[505]

		RABV titers were among *Artibeus planirostris*, 24/46 (52.2%)	
Australia (4 states/territories)	Samples from 656 wild-caught bats and 14 submitted bats from 14 genera and seven families	Evidence of ABLV infection in five of the six families of bats, and three of the four states/territories surveyed	[498]
Australia (western)	649 bat serum samples in western Australia	Antibodies to ABLV detected in 19 individuals across six taxa, with a crude prevalence of 2.9% (95% CI: 1.8‑4.5%) over the two years, including the first records of lyssavirus exposure in *Nyctophilus* spp. and *Falsistrellus mackenziei*	[56]
Bangladesh	288 total serum samples obtained from bats in 9 different taxa	Three of 127 (2.4%) serum samples (all from *Pteropus giganteus*) neutralized ARAV and KHUV	[506]
Cambodia	1,303 bats of 16 spp. were tested	Antibodies were detected by ELISA in 144 (14.7%) of 981 sera, and 30 of 187 samples had neutralizing antibodies against several different lyssaviruses	[507]
England	273 *Eptesicus serotinus* and 363 *Myotis daubentonii* samples were tested by the EBLV-1 or EBLV-2	EBLV-2 antibody estimate was 1.0-4.1% for *M. daubentonii,* while EBLV1 -specific antibodies were	[508]

	specific modified fluorescent antibody neutralization test	detected only in a single *E. serotinus*	
Europe	WCBV and LLEBV screening of sera from *Miniopterus schreibersii* across eight countries	Virus neutralizing antibodies were widespread, with serological evidence and positivity ranging from 3%-67%	[96]
France	*Eptesicus serotinus* maternity colony of ~200 individuals with outbreak of EBLV-1	Active surveillance of the colony by capture of bats showed a high detection rate of neutralising EBLV1 antibodies (~ 50%)	[509]
French Guiana	~1,000 bats from 30 spp.	Seroprevalence to RABV ranged from 0 to 20%, with seropositivity higher in bats with a haematophagous diet	[510]
Grenada	Sera from 111 insectivorous and frugivorous bats belonging to four spp. were tested (52 *Artibeus jamaicensis*, two *Artibeus lituratus*, 33 *Glossophaga longirostris*, 24 *Molossus molossus*)	Antibodies to RABV were detected in the sera of 8/52 (15.4%) *A. jamaicensis* in 4 of the six parishes	[511]
Indian Ocean islands (Anjouan, Mayotte, La Réunion, Mauritius, Mahé, Madagascar)	572 bats, belonging to 22 spp.	97 sera neutralised DUVV, and 42 sera neutralized LBV, but no sample neutralised both DUVV and LBV, while most DUVV-seropositive bats (n = 32/220, 14.5%) also	[512]

		neutralised EBLV-1 (but not RABV)	
Italy	190 samples from bent-winged bats from 2020-21 for reactivity against WCBV	Seasonal seroprevalence varied from 10.9%-42,8%	[61]
Madagascar	*Eidolon dupreanum*	12 of 50 (24%) were positive against LBV	[513]
Nigeria	*Eidolon helvum*	3 of 39 (7.7%) had anti-lyssavirus antibodies	[514]
Philippines (6 different islands)	14 different spp. of insectivorous and frugivorous bats representing five of the six representative families of Chiroptera	22 of 231 (9.5%) samples had evidence of neutralizing antibody against ABLV	[515]
Puerto Rico	218 samples from Antillean fruit-eating bats (*Brachyphylla cavernarum*)	Of 216 bats tested against RABV, 14 (6.5%) had an VNA titer of at least 0.125 IU/ml	[516]
Spain	1223 different captures of *Eptesicus serotinus*, representing 1080 individuals that were sampled and released	Viral circulation was detected in nine colonies either by RT-PCR, anti-EBLV1 serology or both	[517]
Thailand (8 provinces)	932 bats of 11 species	Samples had detectable neutralizing antibodies against ARAV, KHUV, IRKV, or ABLV, all specifically associated with fruit bats, *Pteropus lylei* (15/335, 4.5%) or *Eonycteris spelaea* (1/45, 2.2%)	[518]
Trinidad	383 bat samples representing 21 spp.	Antibodies to RAB*V* detected in 33 samples	[519]

		(8.6%) representing 6 bat spp. (mainly frugivorous), with titers ranging from 0.1 to 19 IU/mL (mean 1.66 IU/mL)	
Vietnam	926 bats collected identified to 25 spp.	Of 789 serum samples tested, 193 (24.5%) were positive for neutralizing antibody activity against lyssaviruses	[520]
